# Supplementary material for: Gm14230 controls Tbc1d24 cytoophidia and neuronal cellular juvenescence
Source: PLoS One. 2021 Apr 22;16(4):e0248517. doi: 10.1371/journal.pone.0248517 (PMC8062039; doi:10.1371/journal.pone.0248517)
Supplement: S1 Fig — Co-immunoprecipitation assay performed in Neuro2a cells overexpressing FLAG-TBC1D24. The pulldown reactions were performed with anti-FLAG or control rabbit antibody. The precipitated protein samples were analyzed by SDS-PAGE followed by immunoblot analysis for Ctps, Impdh and FLAG. IP, immunoprecipitation. (PDF) [file pone.0248517.s001.pdf]

Co-immunoprecipitation analysis

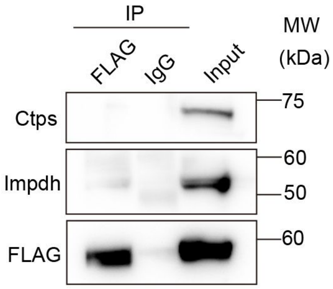

S1 Fig. Co-immunoprecipitation analysis of Tbc1d24 with Ctps or Impdh.
Co-immunoprecipitation assay performed in Neuro2a cells overexpressing FLAG-*TBC1D24*. The pulldown reactions were performed with anti-FLAG or control rabbit antibody. The precipitated protein samples were analyzed by SDS-PAGE followed by immunoblot analysis for Ctps, Impdh and FLAG. IP, immunoprecipitation.
