# Supplementary material for: Gm14230 controls Tbc1d24 cytoophidia and neuronal cellular juvenescence
Source: PLoS One. 2021 Apr 22;16(4):e0248517. doi: 10.1371/journal.pone.0248517 (PMC8062039; doi:10.1371/journal.pone.0248517)
Supplement: S2 Fig — (A) Immunofluorescence analysis of Tbc1d24 in Neuro2a cells treated with 2 μM MPA or control DMSO for 24 hrs followed by washout of MPA. After removing MPA, cells were further cultured for 24 or 48 hrs prior to Tbc1d24 immunostaining. DAPI was used to stain nuclei. Scale bar = 25 μm. (B) Frequency of cells with a cytoophidium. n.s., not significant. **p < 0.01; Student’s t-test. The data were presented as the means ± SEM. (PDF) [file pone.0248517.s002.pdf]

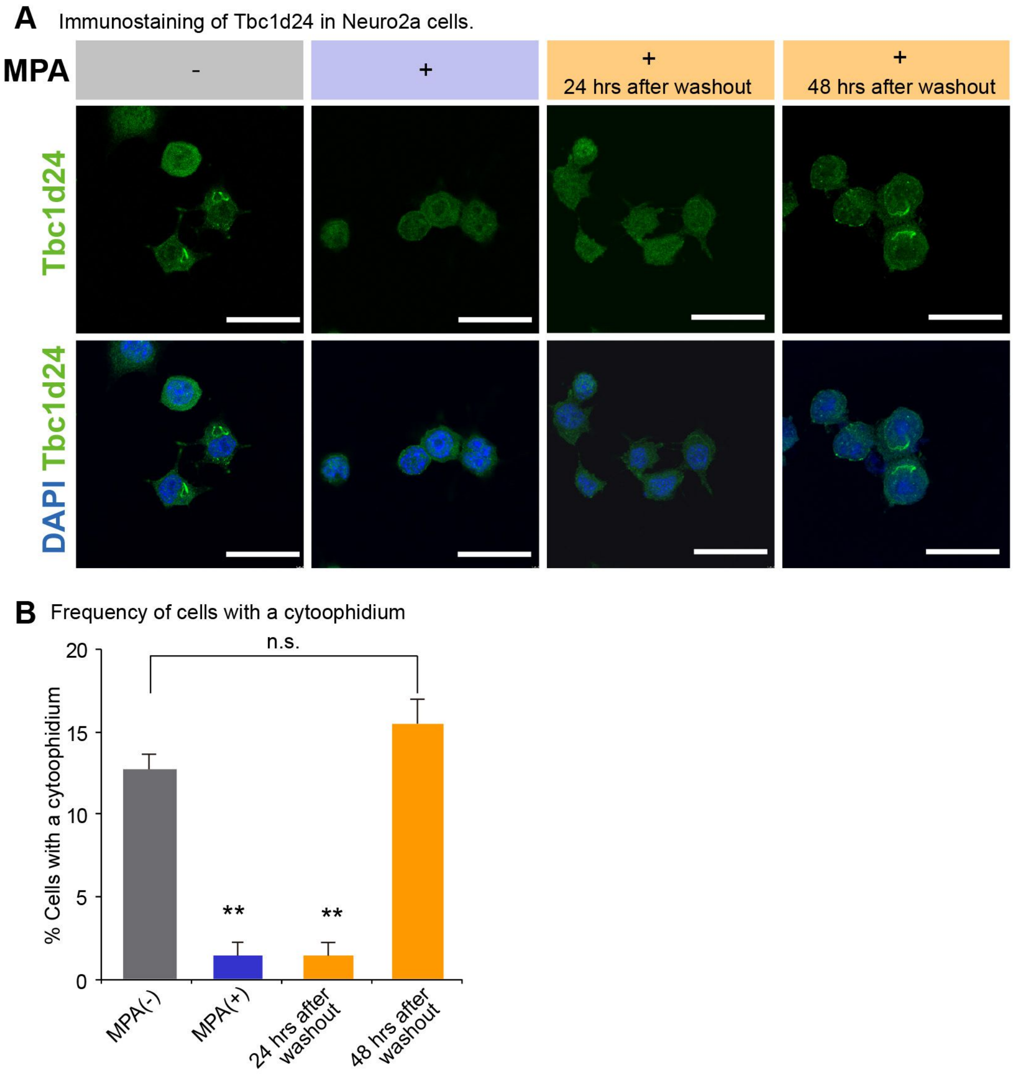

S2 Fig. Reversibility of the Tbc1d24 cytoophidia formation.

(A) Immunofluorescence analysis of Tbc1d24 in Neuro2a cells treated with 2 μM MPA or control DMSO for 24 hrs followed by washout of MPA. After removing MPA, cells were further cultured for 24 or 48 hrs prior to Tbc1d24 immunostaining. DAPI was used to stain nuclei. Scale bar = 25 μm.

(B) Frequency of cells with a cytoophidium. n.s., not significant. $**p < 0.01$; Student's *t*-test. The data were presented as the means ± SEM.
