# Supplementary material for: Gm14230 controls Tbc1d24 cytoophidia and neuronal cellular juvenescence
Source: PLoS One. 2021 Apr 22;16(4):e0248517. doi: 10.1371/journal.pone.0248517 (PMC8062039; doi:10.1371/journal.pone.0248517)
Supplement: S3 Fig — (A) Tbc1d24 immunocytochemistry in Neuro2a cells treated with 2 mM Acivicin or control distilled water for 24 hrs. DAPI was used to stain nuclei. Scale bar = 25 μm. (B) Frequency of Tbc1d24 cytoophidia in Neuro2a cells treated with Acivicin or distilled water. (C) Impdh immunocytochemistry in Neuro2a cells treated with Acivicin or distilled water for 24 hrs. DAPI was used to stain nuclei. Scale bar = 25 μm. (D) Frequency of Impdh cytoophidia in Neuro2a cells treated with Acivicin or distilled water. (E) Ctps immunocytochemistry in Neuro2a cells treated with Acivicin or distilled water for 24 hrs. DAPI was used to stain nuclei. Scale bar = 25 μm. (F) Frequency of Ctps cytoophidia in Neuro2a cells treated with Acivicin or distilled water. n.d., not detected, **p < 0.01; Student’s t-test. The data were presented as the means ± SEM. (PDF) [file pone.0248517.s003.pdf]

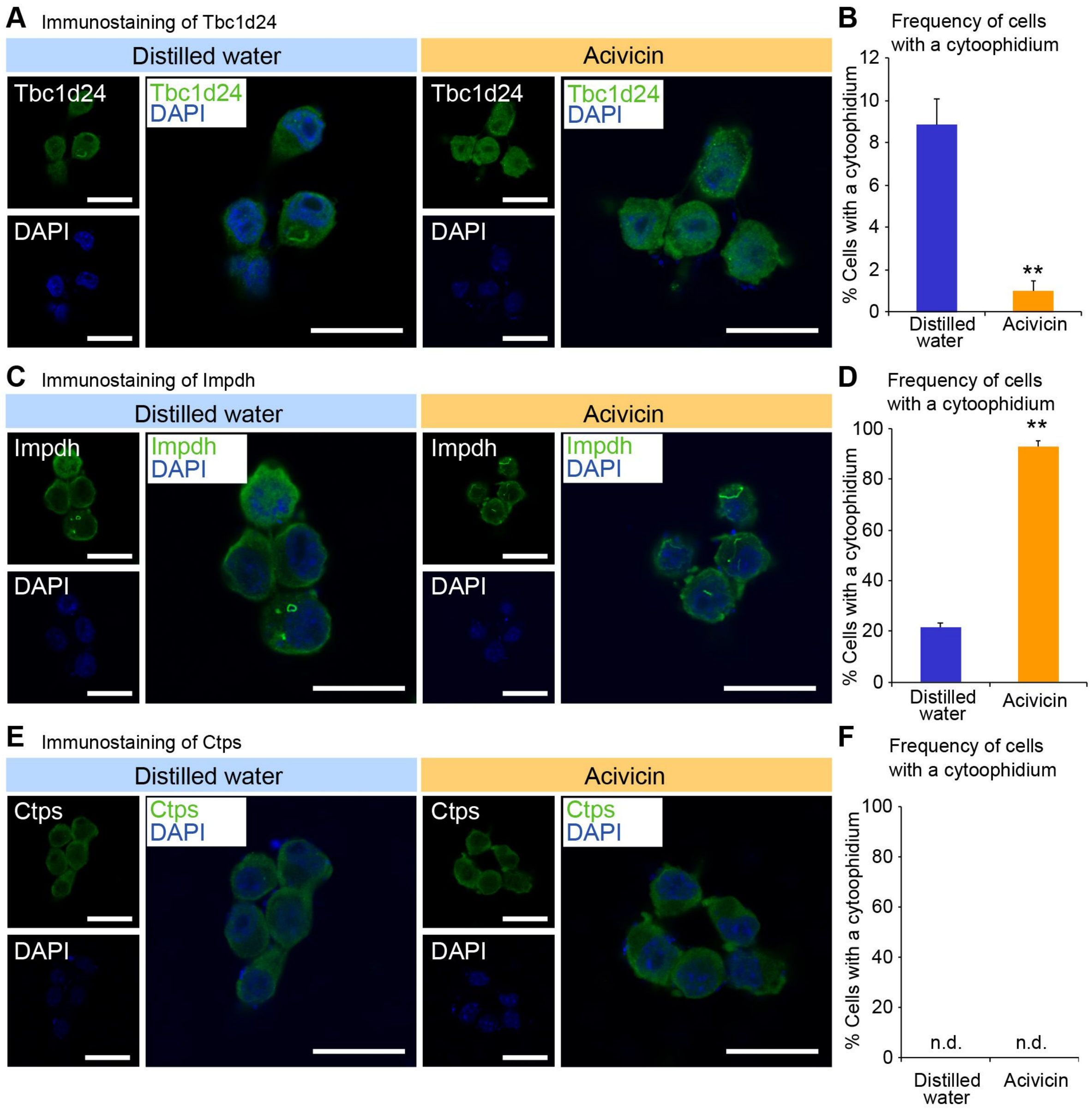

S3 Fig. The Tbc1d24 cytoophidium is distinct from Impdh or Ctps cytoophidium.

(A) Tbc1d24 immunocytochemistry in Neuro2a cells treated with 2 mM Acivicin or control distilled water for 24 hrs. DAPI was used to stain nuclei. Scale bar = 25 μm.

(B) Frequency of Tbc1d24 cytoophidia in Neuro2a cells treated with Acivicin or distilled water.

(C) Impdh immunocytochemistry in Neuro2a cells treated with Acivicin or distilled water for 24 hrs. DAPI was used to stain nuclei. Scale bar = 25 μm.

(D) Frequency of Impdh cytoophidia in Neuro2a cells treated with Acivicin or distilled water.

(E) Ctps immunocytochemistry in Neuro2a cells treated with Acivicin or distilled water for 24 hrs. DAPI was used to stain nuclei. Scale bar = 25 μm.

(F) Frequency of Ctps cytoophidia in Neuro2a cells treated with Acivicin or distilled water.

n.d., not detectet, $^{**}p < 0.01$; Student's *t*-test. The data were presented as the means ± SEM.
