# Supplementary material for: Gm14230 controls Tbc1d24 cytoophidia and neuronal cellular juvenescence
Source: PLoS One. 2021 Apr 22;16(4):e0248517. doi: 10.1371/journal.pone.0248517 (PMC8062039; doi:10.1371/journal.pone.0248517)
Supplement: S4 Fig — (A) Western blot analysis of Neuro2a cells treated with control distilled water, 2 mM DON or 2 mM Acivicin for 24 hrs. Actb was used as loading control. (B) The densitometric analysis for the western blot analysis of Tbc1d24 protein levels in Neuro2a cells treated with DON or Acivicin. The intensity of the bands was quantified and normalized to Actb. The ratios were further normalized to Ctrl treatment. n.s., not significant, Student’s t-test. The data were presented as the means ± SEM. (C) Western blot analysis of Neuro2a cells treated with 2 μM MPA or DMSO for 24 hrs. Actb was used as loading control. (D) The densitometric analysis for the western blot analysis of Tbc1d24 protein levels in Neuro2a cells treated with MPA. The intensity of the bands was quantified and normalized to Actb. The ratios were further normalized to Ctrl treatment. n.s., not significant, Student’s t-test. The data were presented as the means ± SEM. (PDF) [file pone.0248517.s004.pdf]

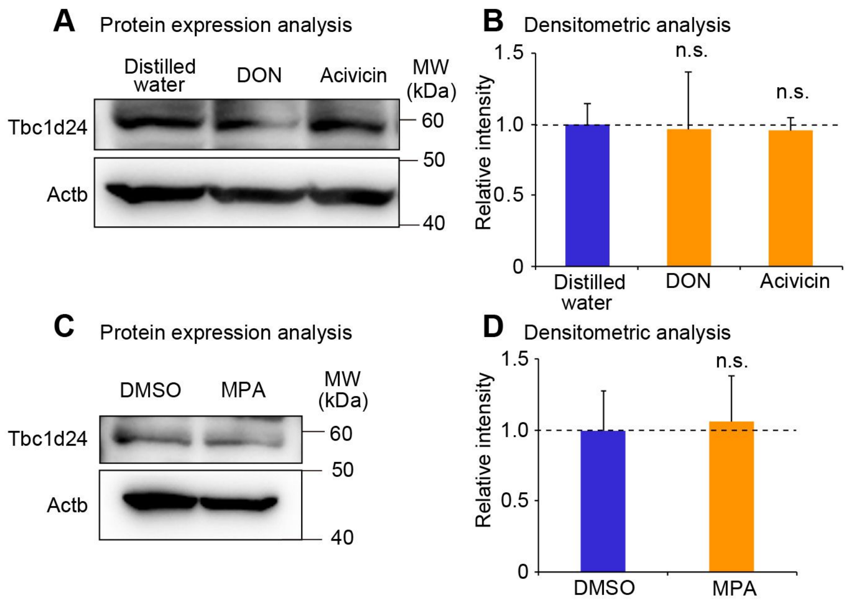

S4 Fig. Tbc1d24 protein levels did not decrease significantly with the treatment with DON, Acivicin or MPA.

(A) Western blot analysis of Neuro2a cells treated with control distilled water, 2 mM DON or 2 mM Acivicin for 24 hrs. Actb was used as loading control.

(B) The densitometric analysis for the western blot analysis of Tbc1d24 protein levels in Neuro2a cells treated with DON or Acivicin. The intensity of the bands was quantified and normalized to Actb. The ratios were further normalized to Ctrl treatment. n.s., not significant, Student's *t*-test. The data were presented as the means ± SEM.

(C) Western blot analysis of Neuro2a cells treated with 2 μM MPA or DMSO for 24 hrs. Actb was used as loading control.

(D) The densitometric analysis for the western blot analysis of Tbc1d24 protein levels in Neuro2a cells treated with MPA. The intensity of the bands was quantified and normalized to Actb. The ratios were further normalized to Ctrl treatment. n.s., not significant, Student's *t*-test. The data were presented as the means ± SEM.
