# Supplementary material for: Gm14230 controls Tbc1d24 cytoophidia and neuronal cellular juvenescence
Source: PLoS One. 2021 Apr 22;16(4):e0248517. doi: 10.1371/journal.pone.0248517 (PMC8062039; doi:10.1371/journal.pone.0248517)
Supplement: S5 Fig — (A) The effect of Tbc1d24 overexpression was examined in siGm14230-induced loss of juvenescence. The appearance of Neuro2a cells transfected with control empty plasmid or TBC1D24 plasmid simultaneously with control siRNA or Gm14230 siRNA. Scale bar = 100 μm. (B) Number of cells per field. The growth was investigated in Neuro2a cells transfected with or control empty plasmid or TBC1D24 plasmid simultaneously with control siRNA or Gm14230 siRNA. **p < 0.01; Student’s t-test. The data were presented as the means ± SEM. (PDF) [file pone.0248517.s005.pdf]

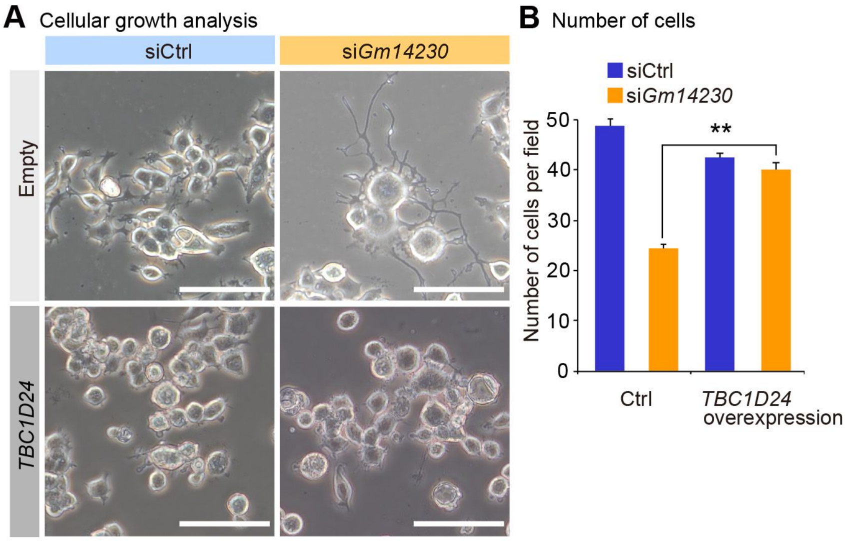

S5 Fig. Forced expression of *Tbc1d24* exerts the protective effect in the loss of cellular juvenescence.

(A) The effect of *Tbc1d24* overexpression was examined in si*Gm14230*-induced loss of juvenescence. The appearance of Neuro2a cells transfected with control empty plasmid or *TBC1D24* plasmid simultaneously with control siRNA or *Gm14230* siRNA. Scale bar = 100 µm.

(B) Number of cells per field. The growth was investigated in Neuro2a cells transfected with or control empty plasmid or *TBC1D24* plasmid simultaneously with control siRNA or *Gm14230* siRNA. ***p* < 0.01; Student's *t*-test. The data were presented as the means ± SEM.
