# Supplementary material for: Gm14230 controls Tbc1d24 cytoophidia and neuronal cellular juvenescence
Source: PLoS One. 2021 Apr 22;16(4):e0248517. doi: 10.1371/journal.pone.0248517 (PMC8062039; doi:10.1371/journal.pone.0248517)
Supplement: S6 Fig — (A) Appearance of Neuro2a cells 48 hrs after the simultaneous knockdown of Gm14230 and Tbc1d24. Indicated siRNAs were transfected in Neuro2a cells for the evaluation of cell growth. Scale bar = 100 μm. (B) Quantification of Neuro2a cells transfected the indicated siRNAs. (C) Sytox blue staining in the same fields as in (A). The gray dots indicated dead cells stained by Sytox blue. (D) Frequency of Sytox blue-positive cells. **p < 0.01; Student’s t-test. The data were presented as the means ± SEM. (PDF) [file pone.0248517.s006.pdf]

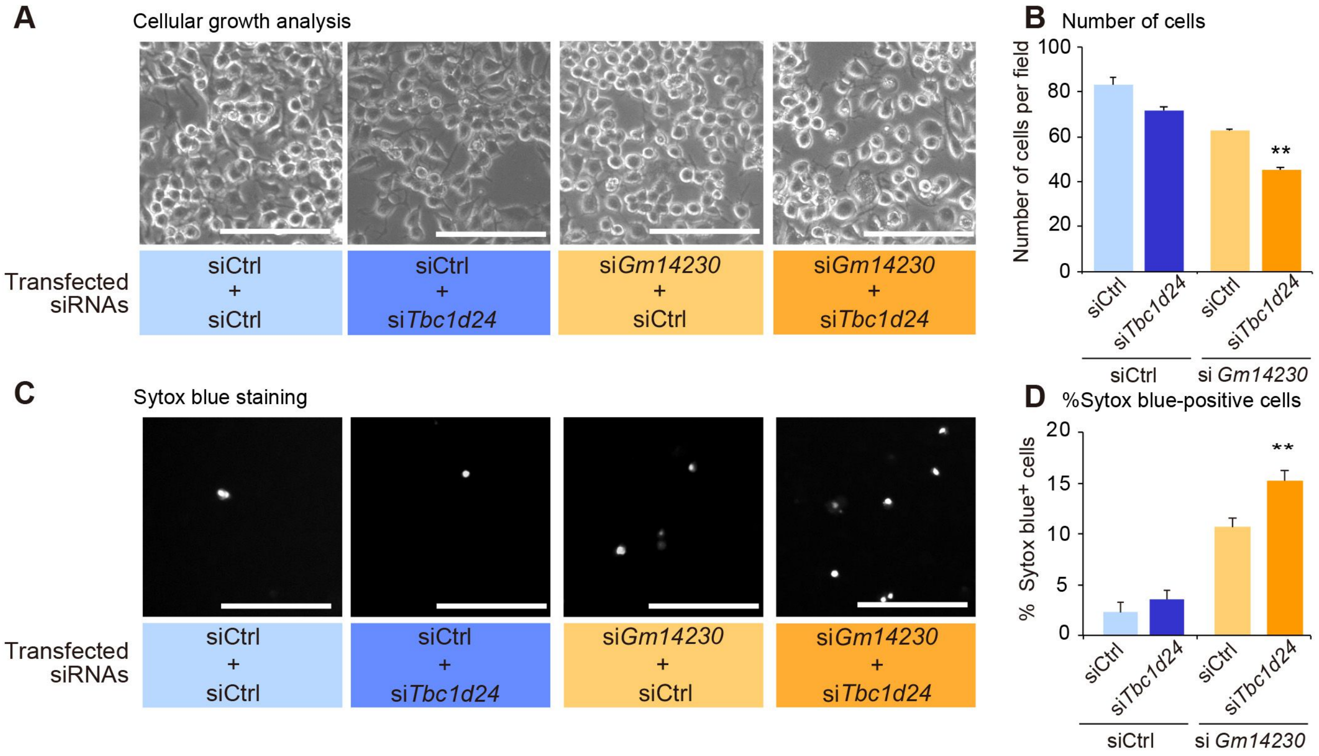

S6 Fig. The protective effect of Tbc1d24 in the *Gm14230*-depletion-induced loss of cellular juvenescence.

(A) Appearance of Neuro2a cells 48 hrs after the simultaneous knockdown of *Gm14230* and *Tbc1d24*. Indicated siRNAs were transfected in Neuro2a cells for the evaluation of cell growth. Scale bar = 100 μm.

(B) Quantification of Neuro2a cells transfected the indicated siRNAs.

(C) Sytox blue staining in the same fields as in (A). The gray dots indicated dead cells stained by Sytox blue.

(D) Frequency of Sytox blue-positive cells.

**p < 0.01; Student's t-test. The data were presented as the means ± SEM.
