# Supplementary material for: Gm14230 controls Tbc1d24 cytoophidia and neuronal cellular juvenescence
Source: PLoS One. 2021 Apr 22;16(4):e0248517. doi: 10.1371/journal.pone.0248517 (PMC8062039; doi:10.1371/journal.pone.0248517)
Supplement: S7 Fig — (A) Tbc1d24 immunocytochemistry in Neuro2a cells treated with zeocin for 0, 48, 72 and 96 hrs. DAPI was used to stain nuclei. Scale bar = 25 μm. (B) Frequency of cells positive for Tbc1d24 cytoophidium in Neuro2a cells treated with zeocin for 0, 48, 72 and 96 hrs. n.s. not significant. **p < 0.01; Student’s t-test. The data were presented as the means ± SEM. (C) Length (μm) of Tbc1d24 cytoophidium in Neuro2a cells treated with zeocin for 0, 48, 72 and 96 hrs. **p < 0.01; Student’s t-test. The data were presented as the means ± SEM. (PDF) [file pone.0248517.s007.pdf]

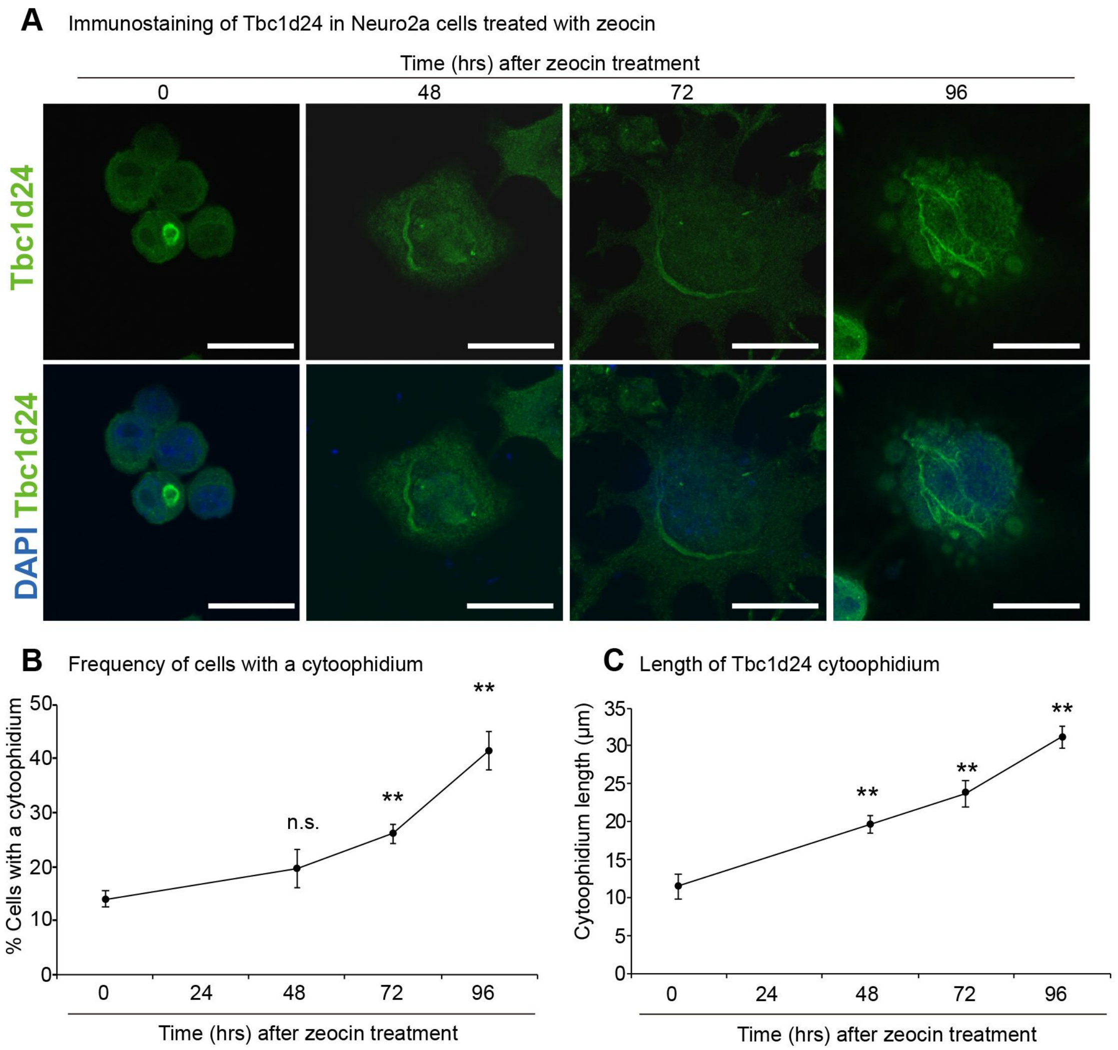

S7 Fig. Tbc1d24 cytoophidia formation is promoted by zeocin induced-cellular senescence in the time-dependent manner.

(A) Tbc1d24 immunocytochemistry in Neuro2a cells treated with zeocin for 0, 48, 72 and 96 hrs. DAPI was used to stain nuclei. Scale bar = 25 μm.

(B) Frequency of cells positive for Tbc1d24 cytoophidium in Neuro2a cells treated with zeocin for 0, 48, 72 and 96 hrs. n.s. not significant. $^{**}p < 0.01$; Student's *t*-test. The data were presented as the means ± SEM.

(C) Length (μm) of Tbc1d24 cytoophidium in Neuro2a cells treated with zeocin for 0, 48, 72 and 96 hrs. $^{**}p < 0.01$; Student's *t*-test. The data were presented as the means ± SEM.
