# Supplementary material for: Gm14230 controls Tbc1d24 cytoophidia and neuronal cellular juvenescence
Source: PLoS One. 2021 Apr 22;16(4):e0248517. doi: 10.1371/journal.pone.0248517 (PMC8062039; doi:10.1371/journal.pone.0248517)
Supplement: S8 Fig — (A) The half-life of Tbc1d24 protein was analyzed by the cycloheximide (CHX) chase assay in Neuro2a cells treated with control distilled water or zeocin for 72 hrs. The cells were treated with CHX 20 μg/ml for 0, 4, 8 and 12 hrs. Actb was used as a loading control. (B) Quantitative analysis of western blots for Tbc1d24 was shown. The intensity of the bands was quantified and normalized to those of Actb. The ratios to Actb were further normalized to 0 hr. *p < 0.05; Student’s t-test. The data were presented as the means ± SEM. (PDF) [file pone.0248517.s008.pdf]

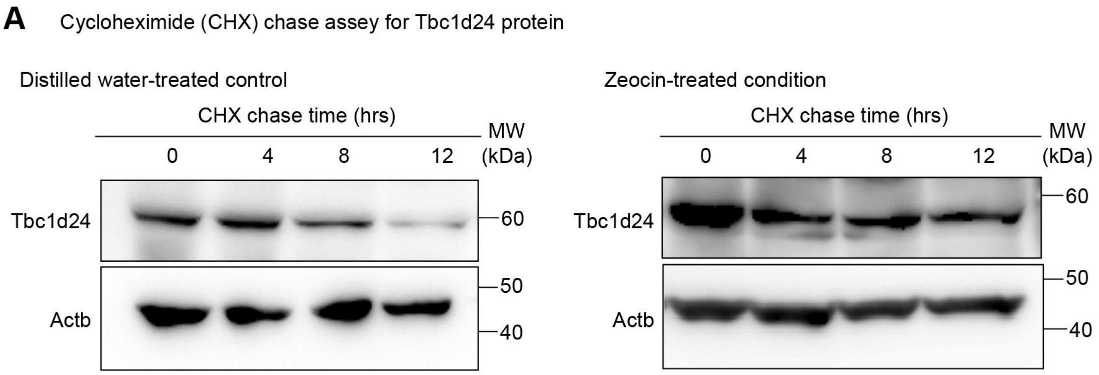

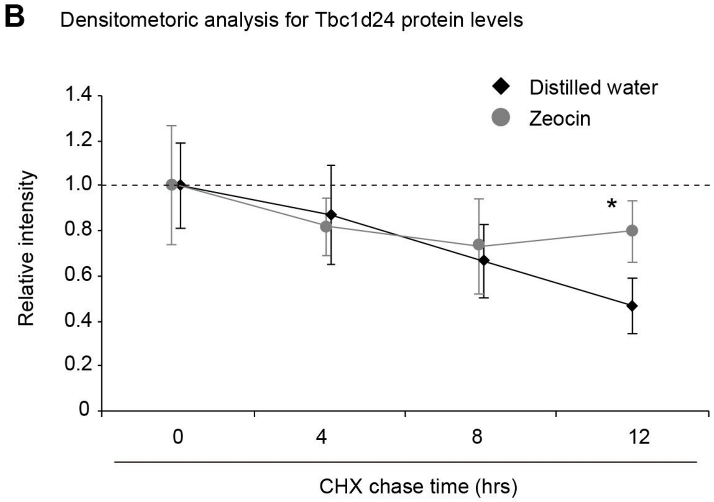

S8 Fig. Tbc1d24 protein is stabilized by cytoophidia formation.

(A) The half-life of Tbc1d24 protein was analyzed by the cycloheximide (CHX) chase assay in Neuro2a cells treated with control distilled water or zeocin for 72 hrs. The cells were treated with CHX 20 μg/ml for 0, 4, 8 and 12 hrs. Actb was used as a loading control.

(B) Quantitative analysis of western blots for Tbc1d24 was shown. The intensity of the bands was quantified and normalized to those of Actb. The ratios to Actb were further normalized to 0 hr. **p* < 0.05 ; Student's *t*-test. The data were presented as the means ± SEM.
