# Supplementary material for: Gm14230 controls Tbc1d24 cytoophidia and neuronal cellular juvenescence
Source: PLoS One. 2021 Apr 22;16(4):e0248517. doi: 10.1371/journal.pone.0248517 (PMC8062039; doi:10.1371/journal.pone.0248517)
Supplement: S9 Fig — (A) The effect of Tbc1d24 overexpression was examined in zeocin-induced cellular senescence. Prior to zeocin treatment, Neuro2a cells were transfected with TBC1D24 or control empty plasmid. The cell appearance was observed to evaluate cell viability. Scale bar = 100 μm. (B) The number of cells per field. The growth was investigated in Neuro2a cells transfected with TBC1D24 or control empty plasmid prior to zeocin treatment. **p < 0.01; Student’s t-test. The data were presented as the means ± SEM. (PDF) [file pone.0248517.s009.pdf]

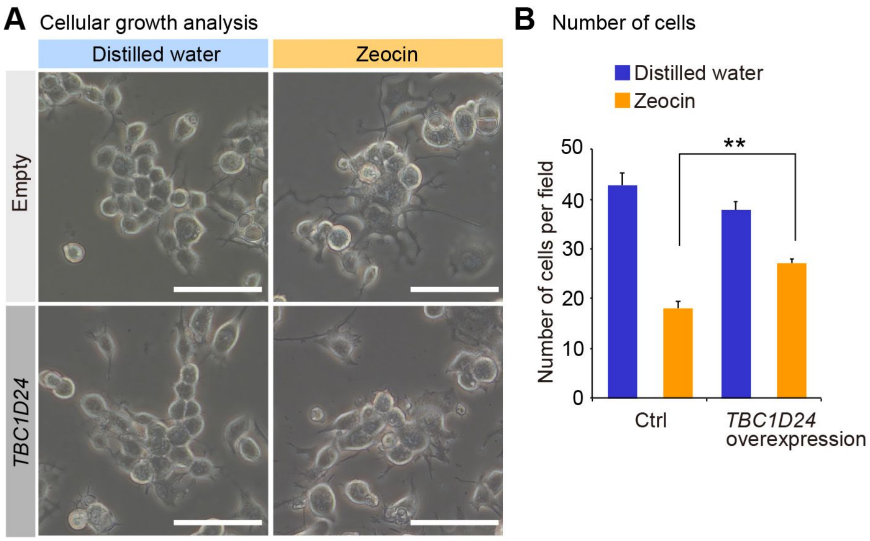

S9 Fig. Forced expression of *Tbc1d24* exerts the protective effect in zeocin-induced cellular senescence.
(A) The effect of *Tbc1d24* overexpression was examined in zeocin-induced cellular senescence. Prior to zeocin treatment, Neuro2a cells were transfected with *TBC1D24* or control empty plasmid. The cell appearance was observed to evaluate cell viability. Scale bar = 100 μm.
(B) The number of cells per field. The growth was investigated in Neuro2a cells transfected with *TBC1D24* or control empty plasmid prior to zeocin treatment. **$p < 0.01$; Student's t-test. The data were presented as the means ± SEM.
