# Supplementary material for: Gm14230 controls Tbc1d24 cytoophidia and neuronal cellular juvenescence
Source: PLoS One. 2021 Apr 22;16(4):e0248517. doi: 10.1371/journal.pone.0248517 (PMC8062039; doi:10.1371/journal.pone.0248517)
Supplement: S10 Fig — (A) GTP-bound Arf6 pulldown assay in Neuro2a cells at 0, 48 and 72 hrs after transfection with Gm14230 siRNA. Total Arf6 levels were examined as loading control. (B) The densitometric analysis of GTP-bound Arf6 pulldown assays. The intensity of the bands was quantified and normalized to those of total Arf6. The ratios to total Arf6 were further normalized to 0 hr. *p < 0.05 and **p < 0.01; Student’s t-test. The data were presented as the means ± SEM. (C) Immunofluorescence analysis of Tbc1d24 in Neuro2a cells at 0, 48 and 72 hrs after transfection with Gm14230 siRNA. DAPI was used to stain nuclei. Scale bar = 25 μm. (D) Frequency of Tbc1d24 cytoophidia positive cells at 0, 48 and 72 hrs after transfection with Gm14230 siRNA. *p < 0.05 and **p < 0.01; Student’s t-test. The data were presented as the means ± SEM. (E) Length (μm) of Tbc1d24 cytoophidium in Neuro2a cells was measured at 0, 48 and 72 hrs after transfection with Gm14230 siRNA. *p < 0.05 and **p < 0.01; Student’s t-test. The data were presented as the means ± SEM. (PDF) [file pone.0248517.s010.pdf]

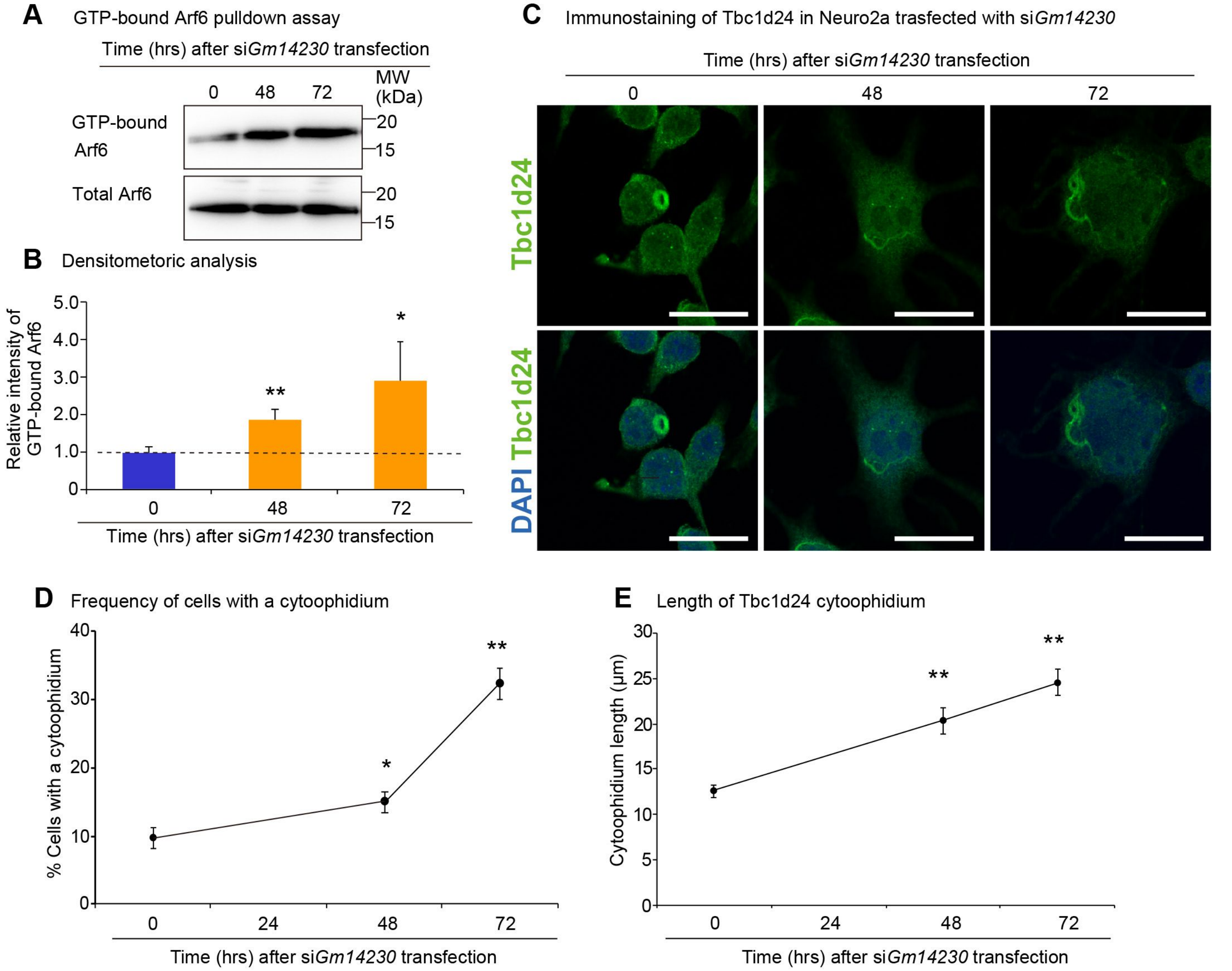

S10 Fig. Correlation of the cytoophidia size and the GAP activity of Tbc1d24.

(A) GTP-bound Arf6 pulldown assay in Neuro2a cells at 0, 48 and 72 hrs after transfection with *Gm14230* siRNA. Total Arf6 levels were examined as loading control.

(B) The densitometric analysis of GTP-bound Arf6 pulldown assays. The intensity of the bands was quantified and normalized to those of total Arf6. The ratios to total Arf6 were further normalized to 0 hr. *$p < 0.05$ and **$p < 0.01$; Student's *t*-test. The data were presented as the means ± SEM.

(C) Immunofluorescence analysis of Tbc1d24 in Neuro2a cells at 0, 48 and 72 hrs after transfection with *Gm14230* siRNA. DAPI was used to stain nuclei. Scale bar = 25 μm.

(D) Frequency of Tbc1d24 cytoophidia positive cells at 0, 48 and 72 hrs after transfection with *Gm14230* siRNA. *$p < 0.05$ and **$p < 0.01$; Student's *t*-test. The data were presented as the means ± SEM.

(E) Length (μm) of Tbc1d24 cytoophidium in Neuro2a cells was measured at 0, 48 and 72 hrs after transfection with *Gm14230* siRNA. *$p < 0.05$ and **$p < 0.01$; Student's *t*-test. The data were presented as the means ± SEM.
