# Supplementary figures and images for: Gm14230 controls Tbc1d24 cytoophidia and neuronal cellular juvenescence
Source: PLoS One. 2021 Apr 22;16(4):e0248517. doi: 10.1371/journal.pone.0248517 (PMC8062039; doi:10.1371/journal.pone.0248517)

**Fig 3**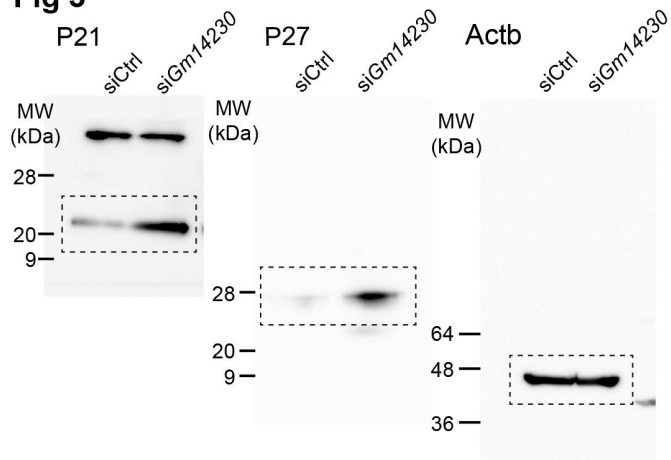**Fig 6 A**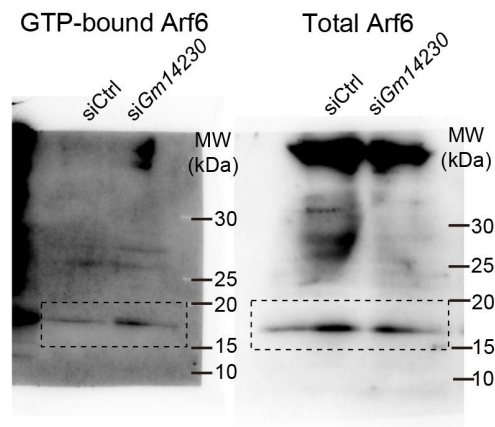**S1A Fig**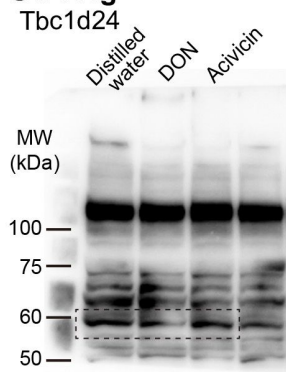**S1C Fig**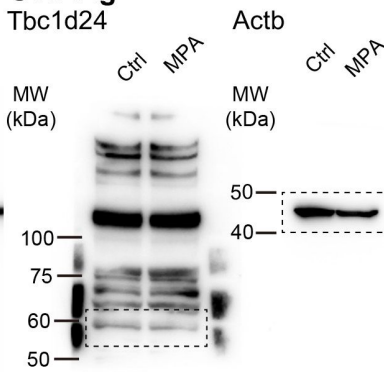**S3 Fig**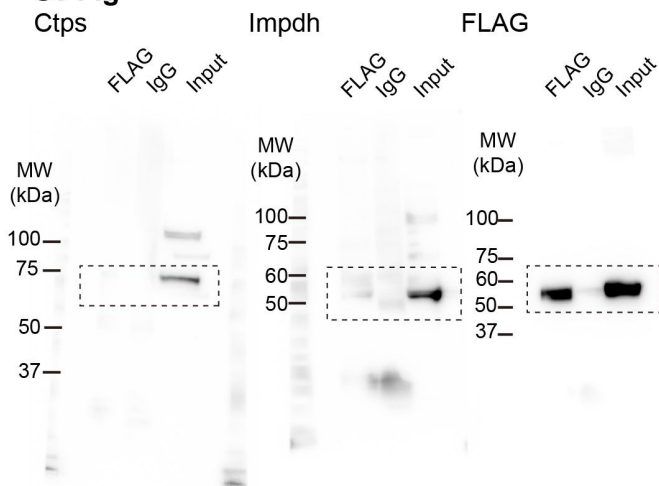**S11 Fig**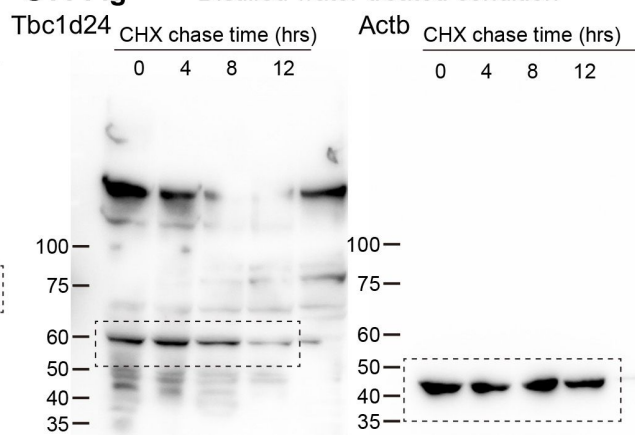**S5A Fig**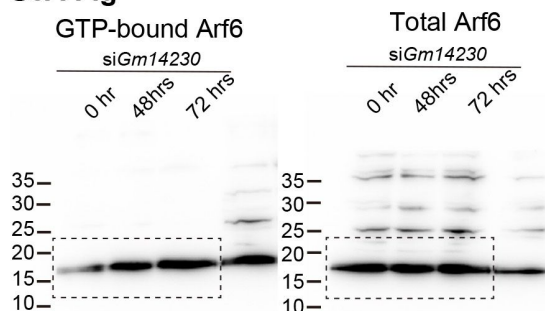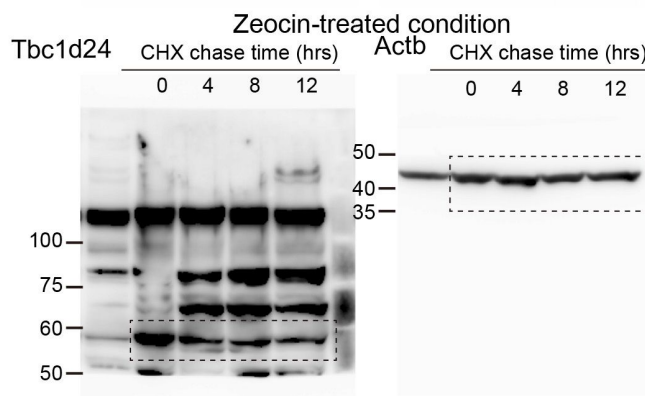**S11 Fig. The uncropped images of western blot.**

Supplement: S1 Raw images — (PDF) [file pone.0248517.s011.pdf]
