# Supplementary material for: Gm14230 controls Tbc1d24 cytoophidia and neuronal cellular juvenescence
Source: PLoS One. 2021 Apr 22;16(4):e0248517. doi: 10.1371/journal.pone.0248517 (PMC8062039; doi:10.1371/journal.pone.0248517)
Supplement: S1 Table — The expression levels of the relevant genes in this study. Shown were the fragments per kilobase of transcript per million fragments sequenced (FPKM) values obtained from the RNA-seq analysis of the mouse cerebral cortex at postnatal day 1 (P1) and P56. (PDF) [file pone.0248517.s012.pdf]

Gene	P1			P56		
	**1**	**2**	**3**	**1**	**2**	**3**
Tbc1d24	8.6	12.0	13.7	5.2	5.4	7.0
Rab35	49.6	41.5	42.0	34.7	32.9	32.9
Arf6	34.4	31.9	27.6	18.1	17.6	18.0
Gm14230	1.0	1.3	1.2	0.1	0.1	0.1
Impdh1	24.6	24.1	24.1	16.5	15.8	17.3
Impdh2	21.0	16.7	16.1	7.7	8.0	7.2
Ctps1	30.2	28.5	29.4	13.1	13.2	13.7
Ctps2	10.8	11.2	10.9	6.6	6.8	7.8

S1 Table. Expression levels of the relevant genes.

The expression levels of the relevant genes in this study. Shown were the fragments per kilobase of transcript per million fragments sequenced (FPKM) values obtained from the RNA-seq analysis of the mouse cerebral cortex at postnatal day 1 (P1) and P56.
