# Supplementary material for: Gm14230 controls Tbc1d24 cytoophidia and neuronal cellular juvenescence
Source: PLoS One. 2021 Apr 22;16(4):e0248517. doi: 10.1371/journal.pone.0248517 (PMC8062039; doi:10.1371/journal.pone.0248517)
Supplement: Attachment — Submitted filename: Response to Reviewers.pdf [file pone.0248517.s014.pdf]

Response to Reviewers

Review Comments to the Author

Reviewer #1: The manuscript entitled "Gm14230 controls Tbc1d24-cytoophidia and neuronal cellular juvenescence" by Morimune et al. studied the formation of Tbc1d24 cytoophidia and its association with Tbc1d24 enzymatic activity and cellular juvenescence. They found in their in vivo and in vitro studies: Tbc1d24 forms cytoophidia in response to the loss of cellular juvenescence caused by depletion of Gm14230, which could serve as a regulatory machinery for Tbc1d24 enzymatic activity. The Tbc1d24 cytoophidia was also found to be quite different from Ctps and Impdh cytoophidia. Overall, the paper is well-written, and the quality of the presented research work is good. Although I think that it is a good candidate for being published in PLOS one, the following questions should be addressed properly.

We appreciate the reviewer for the professional and thoughtful suggestions. The feedbacks and suggestions were really helpful and improved the quality of our manuscript.

1. Whether these cytoophidia proteins interact with each other biochemically? IFS only represents the distribution of the major portion of the proteins, and there is still a possibility of co-existence of the cytoophidia proteins.

We would like to thank you for questioning about the possible interaction of Tbc1d24 with other cytoophidia-forming proteins (Impdh and Ctps). We performed co-immunoprecipitation analysis in Neuro2a cells overexpressing FLAG-tagged TBC1D24 protein. As a result, neither of Ctps nor Impdh showed co-precipitation with TBC1D24, suggesting that TBC1D24 did not interact with Ctps or Impdh protein.

We added this result as **S1 Fig**.

We also added description in Results and Methods as below.

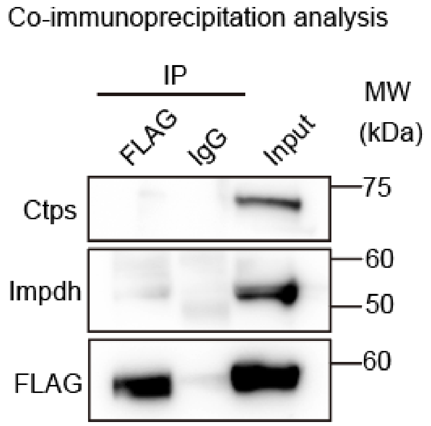

[Figure Legends] Line 691 -.

S1 Fig. Co-immunoprecipitation analysis of Tbc1d24 with Ctps or Impdh.

Co-immunoprecipitation assay performed in Neuro2a cells overexpressing FLAG-*TBC1D24*. The pulldown reactions were performed with anti-FLAG or control rabbit antibody. The precipitated protein samples were analyzed by SDS-PAGE followed by immunoblot analysis for Ctps, Impdh and FLAG. IP, immunoprecipitation. MW, molecular weight.

[Results] Line 275 -.

Co-immunoprecipitation assay with Neuro2a cells expressing FLAG-*Tbc1d24* did not show the appreciable interaction of Tbc1d24 protein with Impdh and Ctps protein (**S1 Fig**).

[Methods] Line 181 -.

Co-Immunoprecipitation assay

To analyze potential interaction of Tbc1d24 with Ctps or Impdh, Neuro2a cells were transfected with FLAG tagged *TBC1D24* plasmid (GenScript). Forty-eight hours after transfection, cells were lysed with NETN buffer containing 20 mM Tris-HCl (pH 8.0), EDTA 1 mM, NaCl 100 mM and 0.5% NP-40, followed by centrifugation at 20,800x g for 15 min at 4°C to remove debris. Protein G Sepharose beads (Invitrogen) were incubated with 2 μg anti-FLAG antibody or control rabbit IgG (Cell Signaling Technology, #2729) for 1 h at RT, and then incubated with the cell lysates extracted from the FLAG-*TBC1D24*-transfected cells for 16 h at 4°C. The beads were then washed with the NETN200 wash buffer containing 20 mM Tris-HCl, 1 mM EDTA, 200 mM NaCl and 0.5% NP-40 four times, followed by elution with Laemmli sample buffer and denaturation at 95°C for 5 min. The samples were analyzed by western blots with anti-Impdh, anti-Ctps and anti-FLAG antibodies.

2. Is cytoophidia protective or toxic to cell survival?

We concluded that the Tbc1d24-cytoophidia was protective against the cellular stresses such as zeocin treatment- (**Fig 5**) or *Gm14230* knockdown-induced loss of cellular juvenescence (**Fig 3**). We clarified this point by adding the statement as below in Discussion.

[Discussion] Line 397 -.

As above, Tbc1d24 cytoophidia may function as the stress-response element and protected cells from zeocin treatment- and *Gm14230*-depletion-induced loss of the cellular juvenescence. This idea was further supported by the findings that *Tbc1d24*-depletion increased cellular toxicity upon *Gm14230* loss (**S6 Fig**) and that *TBC1D24* forced expression rescued the cell toxicity of zeocin treatment (**S9 Fig**).

3. What is the difference between cytoophidia and other aggregations?

We consider that cytoophidia are different from other aggregations, based on the observations of the macrostructure of Tbc1d24 cytoophidia. The structure of Tbc1d24 cytoophidia was similar to those of Impdh, and Ctps cytoophidia, the filamentous structure consisting of enzymatic proteins. These cytoophidia are known to play a biological role: for example, Ctps cytoophidia inhibits Ctps ubiquitination and leads to prolonged half-life of Ctps. Ctps cytoophidia are suggested as reservoir of Ctps. Our data were also shown forming Tbc1d24 cytoophidia prolonged the half-life of Tbc1d24. Tbc1d24 cytoophidia were suggested similar structure and function of Ctps cytoophidia.

We clarified this point by adding the statement as below in Discussion.

[Discussion] Line 402 -.

The filamentous macrostructure of Tbc1d24 cytoophidia were similar to that of Impdh and Ctps cytoophidia, but we demonstrated the Tbc1d24 cytoophidia were distinct from the Impdh and Ctps cytoophidia by a series of experiments.

[Discussion] Line 410 -.

We consider that Tbc1d24 cytoophidia are distinct from mere protein aggregations and function as the reservoir of the enzyme to cope with cellular stresses.

4. Is cytoophidia formation reversible when cell meets stress?

Thank you for pointing out this issue. To test whether Tbc1d24 cytoophidium is reversible, we transiently treated cells with MPA followed by washout. This resulted in the re-formation of cytoophidia with the same frequency as control 48 hours after washout, indicating that cytoophidium formation is reversible.

We added this finding as **S2 Fig.**

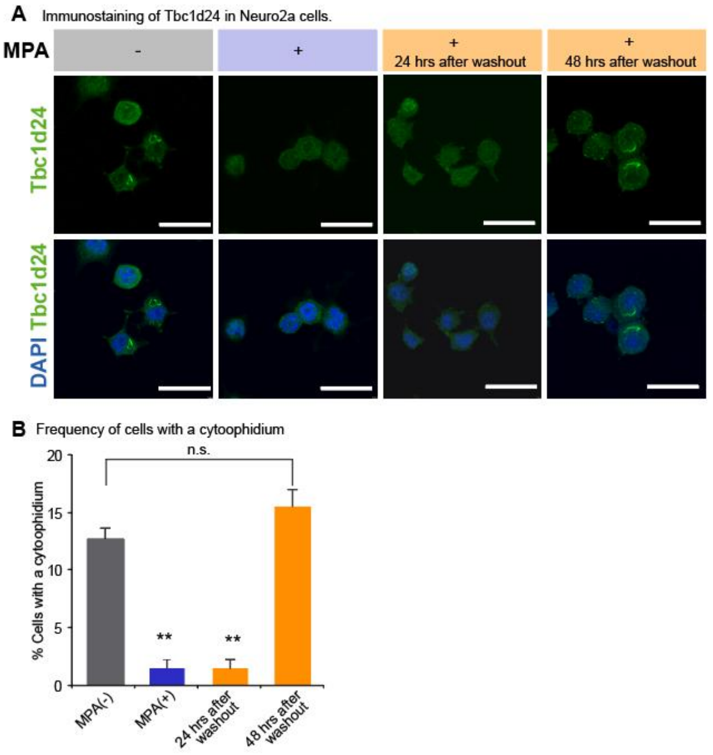

[Figure legends] Line 697 -.

S2 Fig. Reversibility of the Tbc1d24 cytoophidia formation.

(A) Immunofluorescence staining of Tbc1d24 in Neuro2a cells treated with DMSO or 2 μM MPA. After washing out MPA, cells were further cultured for 24 or 48 hrs prior to Tbc1d24 immunostaining. DAPI was used to stain nuclei. Scale bar = 25 μm.

(B) Frequency of cells with a cytoophidium. n.s., not significant. ** $p < 0.01$; Student's *t*-test. The data were presented as the means ± SEM.

[Results] Line 284 -.

Transient treatment of the cells with MPA showed that Tbc1d24 cytoophidia reformed 48 hrs after washing out MPA, revealing the reversible nature of Tbc1d24 cytoophidia formation (**S2 Fig**).

[Methods] Line 219 -.

To assess reversibility of Tbc1d24 cytoophidia formation, Neuro2a cells were treated with 2 μM MPA or dimethyl sulfoxide (DMSO) for 24 hrs before washing out MPA. The cells were then further cultured for 24 or 48 hrs and analyzed for Tbc1d24 cytoophidia by immunocytochemistry described as above.

5. Are these cytoophidia the mixture of different proteins? Can we use Co-IP experiments to prove it?

To test whether Tbc1d24 cytoophidia is the mixture of already known component(s) of cytoophidia, we performed co-immunoprecipitation (Co-IP) experiment with FLAG-Tbc1d24, Impdh and Ctps (as shown above). The result suggested Tbc1d24 cytoophidia were not the mixture of the different protein (Impdh or Ctps).

6. Is there any correlation between the size of Cytoophidia aggregates or their growth rate and the degree of Tbc1d24 enzymatic activity impairment?

We analyzed the correlation of the cytoophidia size and the enzymatic activity. This clarified that the size of cytoophidia negatively correlated with the GTPase activity of Tbc1d24.

We added this finding as **S10 Fig.**

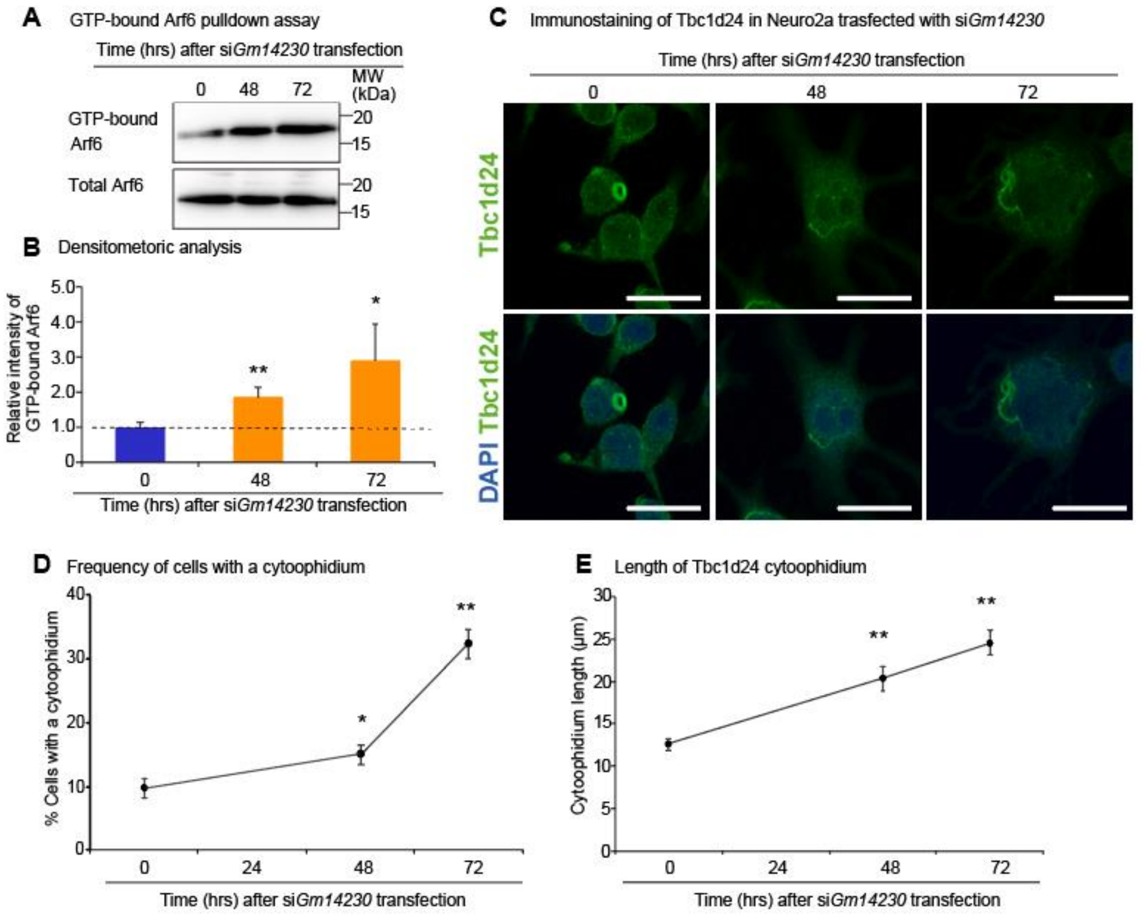

[Figure Legend] Line 779 -.

S10 Fig. Correlation of the cytoophidia size and the GAP activity of Tbc1d24.

(A) GTP-bound Arf6 pulldown assay in Neuro2a cells at 0, 48 and 72 hrs after transfection with *Gm14230* siRNA. Total Arf6 levels were examined as loading control.

(B) The densitometric analysis of GTP-bound Arf6 pulldown assays. The intensity of the bands was quantified and normalized to those of total Arf6. The ratios to total Arf6 were further normalized to 0 hr.

*$p < 0.05$ and **$p < 0.01$; Student's *t*-test. The data were presented as the means ± SEM.

(C) Immunofluorescence analysis of Tbc1d24 in Neuro2a cells at 0, 48 and 72 hrs after transfection with *Gm14230* siRNA. DAPI was used to stain nuclei. Scale bar = 25 μm.

(D) Frequency of Tbc1d24 cytoophidia positive cells at 0, 48 and 72 hrs after transfection with *Gm14230* siRNA. *$p < 0.05$ and **$p < 0.01$; Student's *t*-test. The data were presented as the means ± SEM.

(E) Length (μm) of Tbc1d24 cytoophidium in Neuro2a cells was measured at 0, 48 and 72 hrs after transfection with *Gm14230* siRNA. *$p < 0.05$ and **$p < 0.01$; Student's *t*-test. The data were presented as the means ± SEM.

[Results] Line 362 -.

To assess the correlation between the cytoophidia size and the GAP activity, cells were analyzed for cytoophidia formation and GAP activity in a time course-dependent manner after *Gm14230* depletion. This analysis revealed the time course-dependent impairment of Tbc1d24 GAP activity (**S10A and S10B Fig**) that was accompanied with cytoophidia growth in number (**S10C and S10D Fig**) and size (**S10C and S10E Fig**). These findings suggest that the cytoophidia formation was associated with impaired activity of Tbc1d24.

[Methods] Line 211 -.

The length (μm) of cytoophidia was measured using Fiji [39].

7. Is there any correlation between the size of Cytoophidia aggregates and the degree of cellular senescence?

To test whether there is a correlation between the size of the cytoophidia and the degree of cellular senescence, we tested longer time course in which Neuro2a cells were treated with zeocin to induce cellular senescence for up to 96 hrs. This experiment showed the size of the cytoophidia increased along this longer incubation time, as the cells underwent senescence. Upon this finding, we consider there is a positive correlation of the cytoophidia size and the degree of senescence.

We added this point as **S7 Fig**.

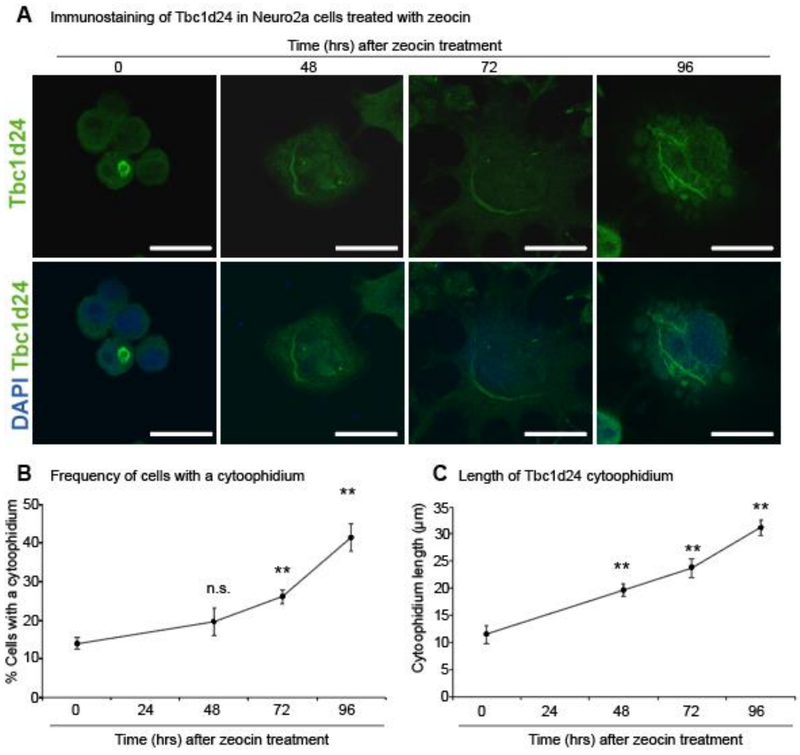

[Figure Legends] Line 752 -

S7 Fig. Tbc1d24 cytoophidia formation is promoted by zeocin induced-cellular senescence in the time-dependent manner.

(A) Tbc1d24 immunocytochemistry in Neuro2a cells treated with zeocin for 0, 48, 72 and 96 hrs. DAPI was used to stain nuclei. Scale bar = 25 μm.

(B) Frequency of cells positive for Tbc1d24 cytoophidium in Neuro2a cells treated with zeocin for 0, 48, 72 and 96 hrs. n.s. not significant. $^{**}p < 0.01$; Student's *t*-test. The data were presented as the means ± SEM.

(C) Length (μm) of Tbc1d24 cytoophidium in Neuro2a cells treated with zeocin for 0, 48, 72 and 96 hrs. $^{**}p < 0.01$; Student's *t*-test. The data were presented as the means ± SEM.

[Results] Line 333 -.

Kinetics for zeocin treatment-induced cytoophidia were analyzed by the time course experiment as follows. Longer incubation with zeocin resulted in more obvious cellular shape changes that were accompanied by more frequent (**S7A and S7B Fig**) and longer (**S7A and S7C Fig**) cytoophidia formation, indicating the positive correlation of the juvenescence loss and cytoophidia formation.

[Methods] Line 211 -.

The length (μm) of cytoophidia was measured using Fiji [39].

Specific questions:

1. There is no figure as 1E.

Thank you for your pointing out this mistake of skipping 1 "E". We corrected this error.

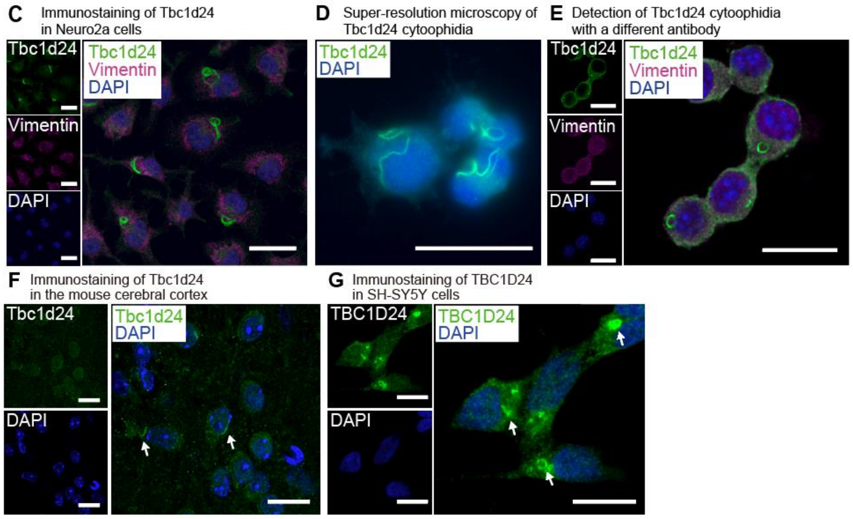

2. What are the MPA, DON, and Acivicin? Authors should include some background information.

We added the statements that provide the background information of DON, Acivicin and MPA in the **Results** section where the compounds were used.

[Results] Line 279 -.

DON and Acivicin are glutamine analogs and inhibit glutamine-dependent enzymes [42]. Both are strong inducer of Impdh and Ctps cytoophidia ([22], [43]). MPA reversibly inhibits Impdh activity that converts IMP to guanosine monophosphate (GMP) [44]. MPA induces Impdh cytoophidia [43].

[Reference] Line 572 -.

42. Hofer A, Steverding D, Chabes A, Brun R, Thelander L. Trypanosoma brucei CTP synthetase: A target for the treatment of African sleeping sickness. Proc Natl Acad Sci U S A. 2001;98: 6412–6416. doi:10.1073/pnas.111139498
43. Gunter JH, Thomas EC, Lengefeld N, Kruger SJ, Worton L, Gardiner EM, et al. Characterisation of inosine monophosphate dehydrogenase expression during retinal development: Differences between variants and isoforms. Int J Biochem Cell Biol. 2008;40: 1716–1728. doi:10.1016/j.biocel.2007.12.018
44. Ji YS, Gu JJ, Makhov AM, Griffith JD, Mitchell BS. Regulation of the interaction of inosine monophosphate dehydrogenase with mycophenolic acid by GTP. J Biol Chem. 2006;281: 206–212. doi:10.1074/jbc.M507056200

3. If we overexpress Tbc1d24 in cells, what will happen? Does it increase the resistance to cellular senescence and protect the cells from external stress?

We performed the overexpression of *TBC1D24* in Neuro2a cells. It increased the resistance to the cellular senescence as shown by the cell proliferation activity. We added this finding as **S5 Fig** and **S9 Fig**.

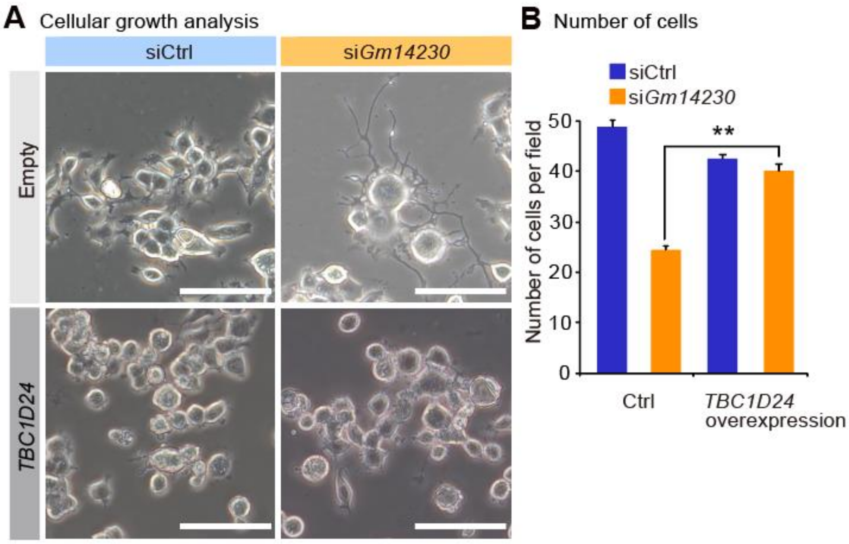

[Figure Legends] Line 732 -.

S5 Fig. Forced expression of *Tbc1d24* exerts the protective effect in the loss of cellular juvenescence.

(A) The effect of *Tbc1d24* overexpression was examined in si*Gm14230*-induced loss of juvenescence. The appearance of Neuro2a cells transfected with control empty plasmid or T*BC1D24* plasmid simultaneously with control siRNA or *Gm14230* siRNA. Scale bar = 100 μm.

(B) Number of cells per field. The growth was investigated in Neuro2a cells transfected with or control empty plasmid or *TBC1D24* plasmid simultaneously with control siRNA or *Gm14230* siRNA. **$p < 0.01$; Student's *t*-test. The data were presented as the means ± SEM.

[Results] Line 316 -.

Inspired by the finding that cytoophidia formation was induced by the loss of cellular juvenescence, we next asked the biological role of cytoophidia in the juvenescence-losing cells. Forced expression of Tbc1d24 ameliorated the growth impairment by *Gm14230* depletion, implying the role of Tbc1d24 to resist the progression of the loss of cellular juvenescence (**S5 Fig**).

[Methods] Line 223 -.

For the assessment of a protective effect of Tbc1d24 in loss of cellular juvenescence, Neuro2a cells were transfected simultaneously with *Gm14230* (or control) siRNA and pcDNA3.1-FLAG-*TBC1D24* (or empty) plasmid. Cell growth were analyzed 48 hrs after the transfection.

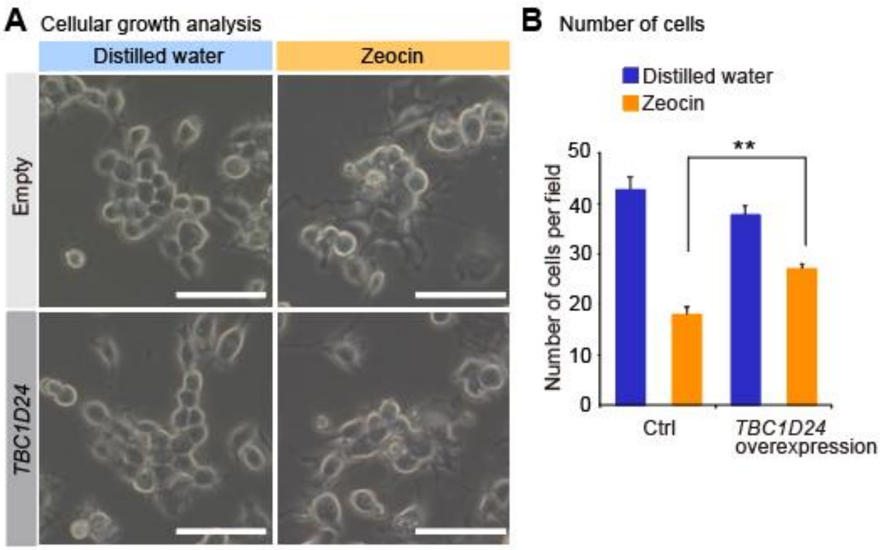

[Figure Legends] Line 770 -.

S9 Fig. Forced expression of *Tbc1d24* exerts the protective effect in zeocin-induced cellular senescence.

(A) The effect of *Tbc1d24* overexpression was examined in zeocin-induced cellular senescence. Prior to zeocin treatment, Neuro2a cells were transfected with *TBC1D24* or control empty plasmid. The cell appearance was observed to evaluate cell viability. Scale bar = 100 µm.
(B) The number of cells per field. The growth was investigated in Neuro2a cells transfected with *TBC1D24* or control empty plasmid prior to zeocin treatment. **$p < 0.01$; Student's *t*-test. The data were presented as the means ± SEM.

[Results] Line 342 -.

To assess a resistive role of Tbc1d24 in zeocin-induced juvenescence loss, we first tested the effect of *Tbc1d24* forced expression. The transfection of *Tbc1d24* alleviated cellular growth impairment and morphological changes induced by zeocin treatment, indicating Tbc1d24 plays a protective role against the cytotoxicity provoked by zeocin-induced juvenescence loss (**S9 Fig**).

[Methods] Line 226 -.

To assess the effect of forced expression of *Tbc1d24* in zeocin-induced cellular juvenescence loss, Neuro2a cells were transfected with pcDNA3.1-FLAG-*TBC1D24* plasmid or control empty pcDNA3.1 plasmid. Twenty-four hrs later, the cells were treated with zeocin at 200 µg/ml or distilled water for 72 hrs for the assessment of cell growth.

4. There are no caption and figure legend for figure 1 H.

Thank you for pointing out this mistake. We corrected 1H as 1 "G".

Thank you for giving us the precious opportunity to significantly improve our paper and refine the research constructively.

Reviewer #2: This is a truly exciting paper with a number of provocative conclusions. However, there are a number of concerns that temper enthusiasm. Some annoying aspects of the paper that impairs the review. Figure legends are split into multiple locations within the text of the paper.

Thank you for giving us this advice. We checked the manuscript and rearranged the Figure legends thoroughly.

The supplement figures are not very large and can be easily incorporated within the figures, particularly for Fig S2.

We moved Supplementary Figures to the main figures as below.

1) The densitometry of P21 and P27 (originally shown as S11 Fig) were moved to **Fig 3**.
2) S2 Fig of previous manuscript was moved to **S11 Fig**.
3) The densitometry of GTP-bound Arf6 pulldown assay was added to **Fig 6B**.

No statistics are provided for the Western blot studies.

To show the results of statistical analyses for the Western blot studies, we added the densitometric analyses in **Fig 3G, Fig 6B, S4B Fig, S4D Fig, S8B Fig and S10B Fig**.

Fig 3G

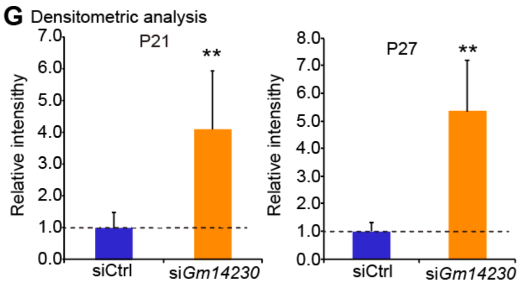

Fig 6B

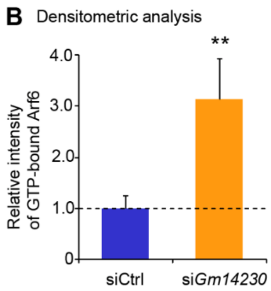

S4B Fig

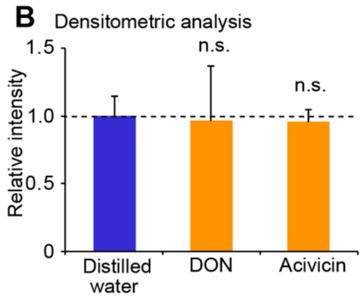

S4D Fig

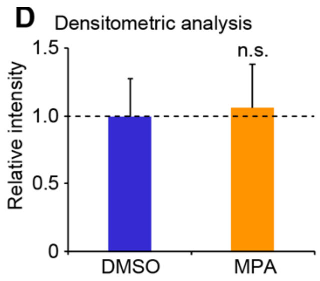

S8B Fig

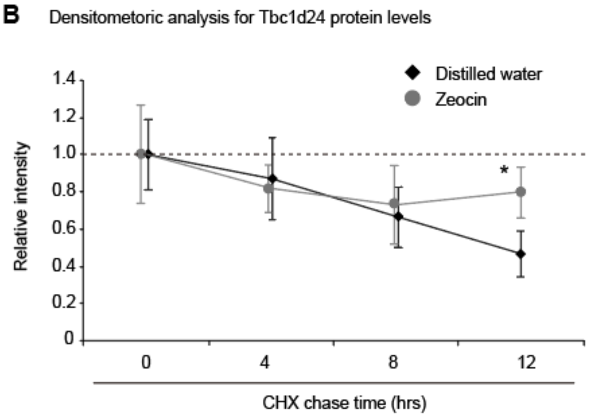

S10B Fig

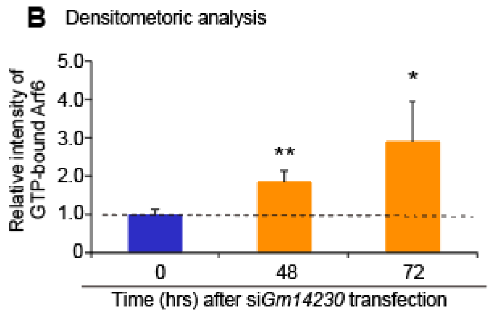

The first is that the paper fails to describe the gene changes in the Juvenility-linked transcriptome assay as a table. This table can be in the form of supplementary data.

We would like to thank you for this suggestion. In a previous study, we performed a comprehensive transcriptome analysis of the juvenility-associated transcriptome changes (Jam et al., **Scientific Reports**, 2018). The next-generation sequencing dataset was deposited to DRA public database with the accession number DRA007101. We would like to add **S1 Table** that shows the expression levels (FPKM values) of juvenile-expressed genes as shown below. We added this result as **S1 Table**.

gene name	P1			P56		
	1	2	3	1	2	3
Tbc1d24	8.6	12.0	13.7	5.2	5.4	7.0
Rab35	49.6	41.5	42.0	34.7	32.9	32.9
Arf6	34.4	31.9	27.6	18.1	17.6	18.0
Gm14230	1.0	1.3	1.2	0.1	0.1	0.1
Impdh1	24.6	24.1	24.1	16.5	15.8	17.3
Impdh2	21.0	16.7	16.1	7.7	8.0	7.2
Ctps1	30.2	28.5	29.4	13.1	13.2	13.7
Ctps2	10.8	11.2	10.9	6.6	6.8	7.8

[Figure Legends] Line 797 -.

S1 Table. Expression of the relevant genes.

The expression levels as fragments per kilobase of transcript per million fragments sequenced (FPKM) in RNA-seq of mouse brain at postnatal day 1 (P1) and P56.

[Results] Line 272 -.

The expression levels of the cytoophidia-related genes *Impdh* and *Ctps* were also higher in juvenile than in adult (**S1 Table**).

[Methods] Line 235 -.

Gene expression analysis in mouse cerebral cortexes

Tbc1d24 expression in mouse cerebral cortexes at postnatal day 1 (P1) and P56 was analyzed by the strand-specific RNA-seq dataset we previously deposited with the accession number DRA009510. The RNA-seq data were processed using BioLinux8 [40]. The expression was visualized using Integrative Genomic Viewer (http://software.broadinstitute.org/software/igv/). Gene expression levels show fragments per kilobase of transcript per million fragments sequenced (FPKM) at P1 and P56.

[Reference] Line 567 -.

40. Field D, Tiwari B, Booth T, Houten S, Swan D, Bertrand N, et al. Open software for biologists: From famine to feast. Nat Biotechnol. 2006;24: 801–803. doi:10.1038/nbt0706-801

The cytoophidia findings in Fig 2 raise several questions. Why is there a strong drop in Tbc1d24 cytoophidia with DON, MPA, Acivicin. Is this reversible?

To examine the potential mechanism underlying the strong drop in Tbc1d24 cytoophidia after the treatment with DON, MPA and Acivicin, we performed western blot analysis to investigate the protein levels of Tbc1d24. As shown below in **S4 Fig**, Tbc1d24 protein levels were not altered significantly by the treatment with DON, MPA and Acivicin, indicating the strong drop in cytoophidia formation was not due to the suppression of Tbc1d24 expression. We added this finding as **S4 Fig**.

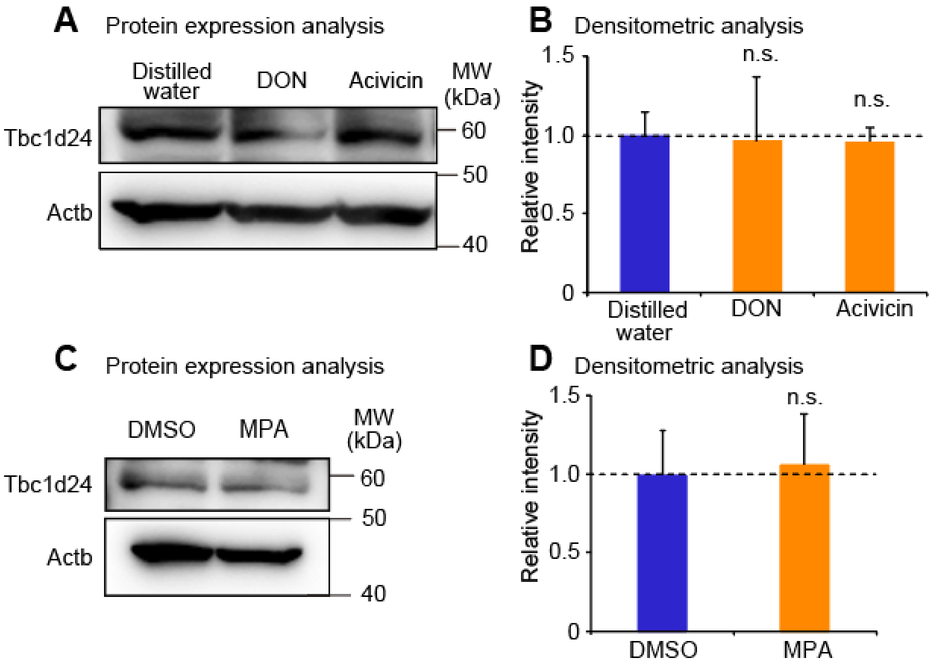

Also, to assess the reversibility of Tbc1d24 cytoophidia formation, we performed transient treatment of cells with MPA. The Neuro2a cells were treated with MPA for 24 hours followed by washing out of MPA. This resulted in the reversible formation of the cytoophidia at 48 hours after the washout, leading us to the finding of the reversibility of Tbc1d24 cytoophidia.

We added this result as **S2 Fig**.

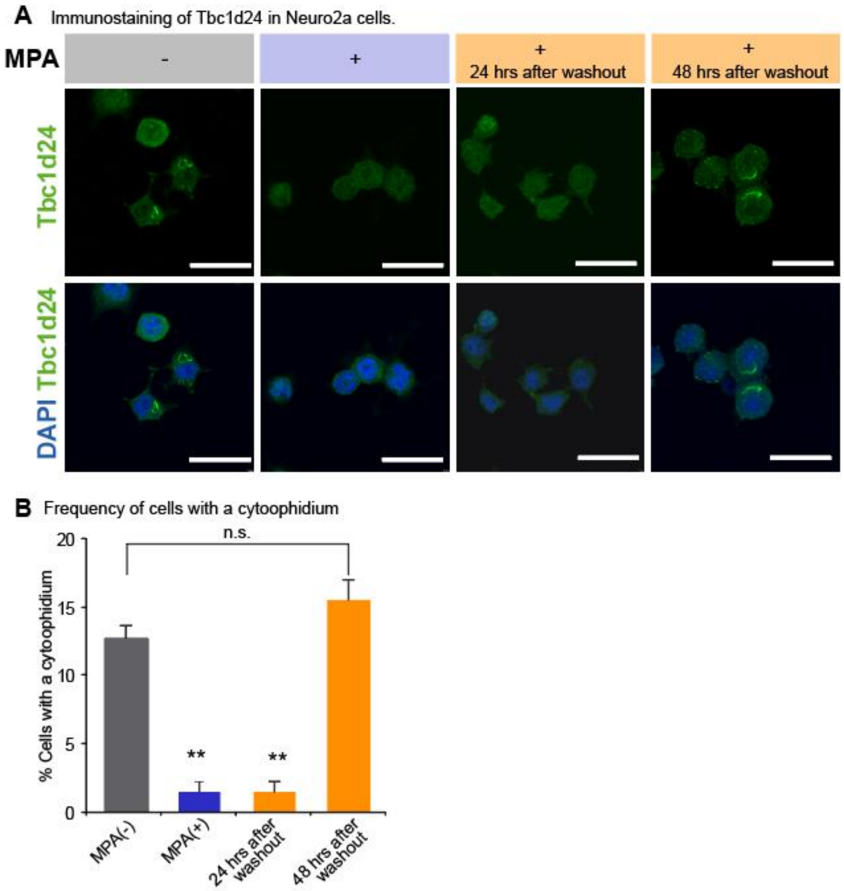

[Figure legends] Line 697 -.

S2 Fig. Reversibility of the Tbc1d24 cytoophidia formation.

(A) Immunofluorescence staining of Tbc1d24 in Neuro2a cells treated with 2 μM MPA or control DMSO for 24 hrs followed by washout of MPA. After removing MPA, cells were further cultured for 24 or 48 hrs prior to Tbc1d24 immunostaining. DAPI was used to stain nuclei. Scale bar = 25 μm.

(B) Frequency of cells with a cytoophidium. n.s., not significant. $^{**}p < 0.01$; Student's *t*-test. The data were presented as the means ± SEM.

[Results] Line 284 -.

Transient treatment of the cells with MPA showed that Tbc1d24 cytoophidia reformed 48 hrs after washing out MPA, revealing the reversible nature of Tbc1d24 cytoophidia formation (**S2 Fig**).

[Methods] Line 219 -.

To assess reversibility of Tbc1d24 cytoophidia formation, Neuro2a cells were treated with 2 μM MPA or dimethyl sulfoxide (DMSO) for 24 hrs before washing out MPA. The cells were then further cultured for 24 or 48 hrs and analyzed for Tbc1d24 cytoophidia by immunocytochemistry described as above.

What happens to levels of Tbc1d24 protein with these treatments? It is useful to show western blots or other protein quantification methods given that this protein is accumulated while the RNA appears to be dropping.

We performed Tbc1d24 western blot analysis to examine the protein levels after treatment with DON, Acivicin or MPA. As a result, the Tbc1d24 protein levels did not show the sharp drop after the treatment, indicating the suppression of cytoophidia formation was not due to the decrease of Tbc1d24 protein.

We added this result as **S4 Fig**.

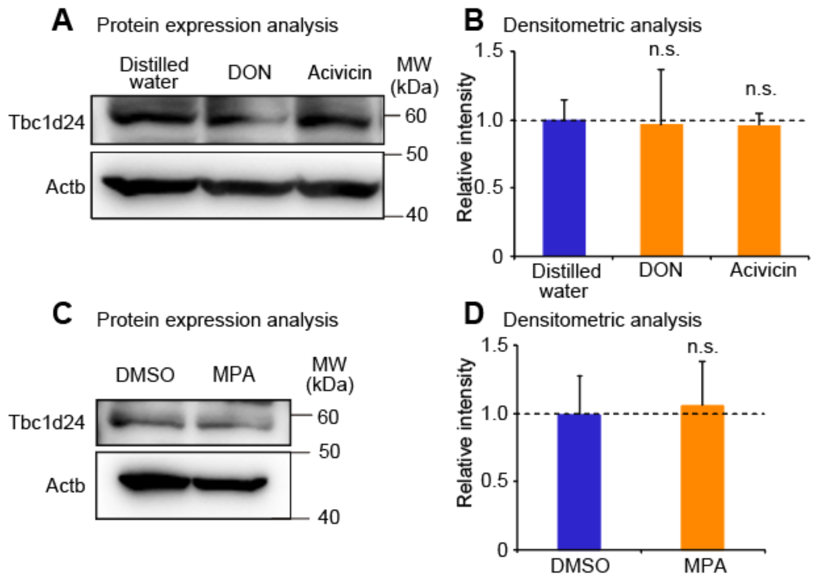

[Figure Legends] Line 717 -.

S4 Fig. Tbc1d24 protein levels did not decrease significantly with the treatment with DON, Acivicin or MPA.

(A) Western blot analysis of Neuro2a cells treated with control distilled water, 2 mM DON or 2 mM Acivicin for 24 hrs. Actb was used as loading control.

(B) The densitometric analysis for the western blot analysis of Tbc1d24 protein levels in Neuro2a cells treated with DON or Acivicin. The intensity of the bands was quantified and normalized to Actb. The ratios were further normalized to Ctrl treatment. n.s., not significant, Student's *t*-test. The data were presented as the means ± SEM.

(C) Western blot analysis of Neuro2a cells treated with 2 μM MPA or DMSO for 24 hrs. Actb was used as loading control.

(D) The densitometric analysis for the western blot analysis of Tbc1d24 protein levels in Neuro2a cells treated with MPA. The intensity of the bands was quantified and normalized to Actb. The ratios were further normalized to Ctrl treatment. n.s., not significant, Student's *t*-test. The data were presented as the means ± SEM.

[Results] Line 292 -.

To examine whether Tbc1d24 protein expression was suppressed by the compounds, we performed western blot analysis that showed Tbc1d24 protein was not decreased significantly by these treatments, indicating that the decrease of cytoophidia was not due to suppression of Tbc1d24 protein (**S4 Fig**).

It is important to demonstrate that the treatments can actually induce cytoophidia with Impdh or Ctps under these conditions.

We performed immunocytochemistry with Impdh and Ctps after treatment with Acivicin. The Acivicin treatment increased the Impdh cytoophidia, confirming our experiment condition was suitable for the cytoophidia analysis. Ctps, however, was not detected in Neuro2a cells in any conditions.

We added this result as **S3 Fig**.

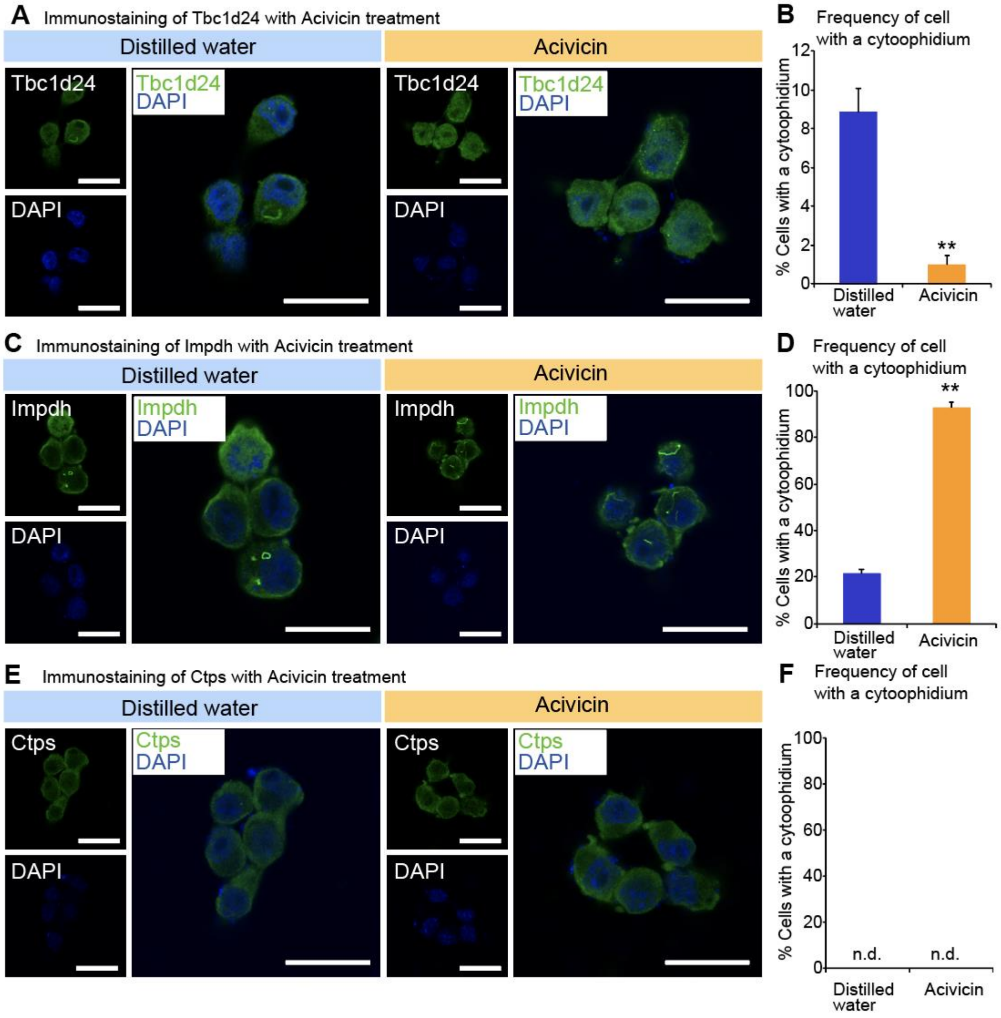

[Figure Legends] Line 705 -.

S3 Fig. The Tbc1d24 cytoophidium is distinct from Impdh or Ctps cytoophidium.

(A) Tbc1d24 immunocytochemistry in Neuro2a cells treated with 2 mM Acivicin or control distilled water for 24 hrs. DAPI was used to stain nuclei. Scale bar = 25 μm.

(B) Frequency of Tbc1d24 cytoophidia in Neuro2a cells treated with Acivicin or distilled water.

(C) Impdh immunocytochemistry in Neuro2a cells treated with Acivicin or distilled water for 24 hrs. DAPI was used to stain nuclei. Scale bar = 25 μm.

(D) Frequency of Impdh cytoophidia in Neuro2a cells treated with Acivicin or distilled water.

(E) Ctps immunocytochemistry in Neuro2a cells treated with Acivicin or distilled water for 24 hrs. DAPI was used to stain nuclei. Scale bar = 25 μm.

(F) Frequency of Ctps cytoophidia in Neuro2a cells treated with Acivicin or distilled water.

n.s., not significant, **$p < 0.01$; Student's *t*-test. The data were presented as the means ± SEM.

[Results] Line 286 -.

Acivicin treatment also resulted in significant suppression of Tbc1d24 cytoophidia (**Figs 2G** and **2H**), making a contrast to Impdh cytoophidia that were markedly induced (**S3C and S3D Fig**).

[Methods] Line 215 -.

To assess the Acivicin-responsibility of Tbc1d24 cytoophidia, Neuro2a cells were treated with 2mM Acivicin or control distilled water for 24 hrs. Acivicin is a known inducer of Impdh and Ctps cytoophidia. Immunofluorescence analyses for Tbc1d24, Impdh and Ctps were conducted to assess the influences on cytoophidia formation.

An age-dependent time course of Tbc1d24 accumulation as filaments will be useful.

We first performed immunohistochemistry in old mice (45 weeks old). But unfortunately, we could not detect Tbc1d24 cytoophidia very clearly probably because of the Tbc1d24 expression was dominant in juvenile and significantly suppressed in adult.

Therefore, we performed the time-course experiment with the Neuro2a cells in which zeocin treatment induced the time-dependent cellular senescence. As a result, the cytoophidia showed time-dependent accumulation suggesting the cytoophidia formation was age-dependent.

We added this result as **S7 Fig.**

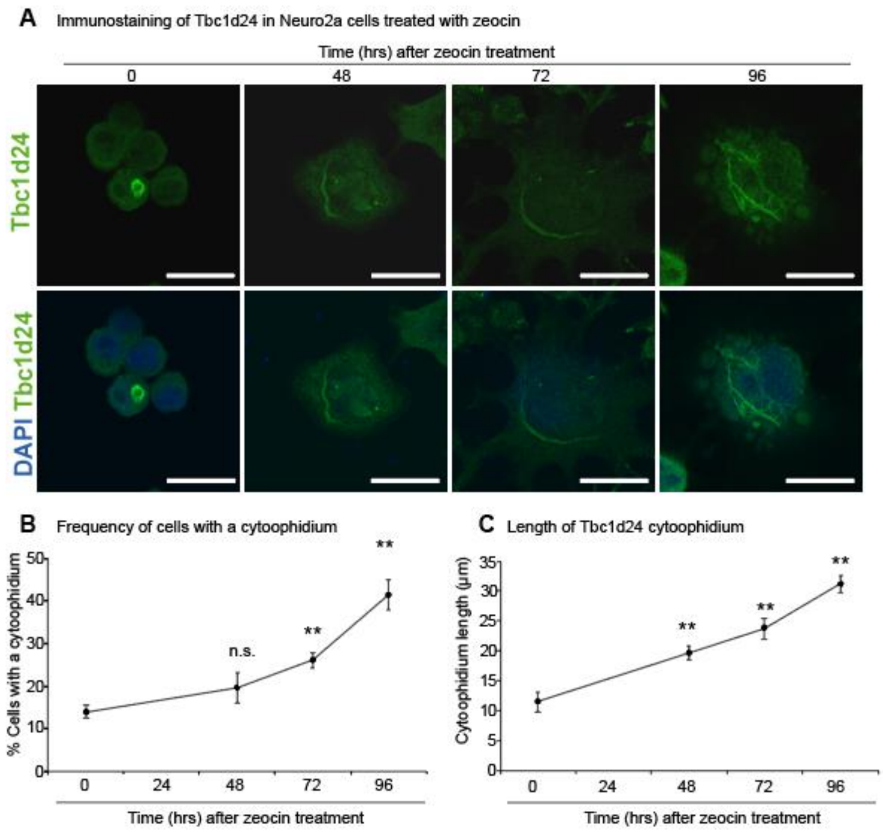

[Figure Legends] Line 752 -

S7 Fig. Tbc1d24 cytoophidia formation is promoted by zeocin induced-cellular senescence in the time-dependent manner.

(A) Tbc1d24 immunocytochemistry in Neuro2a cells treated with zeocin for 0, 48, 72 and 96 hrs. DAPI was used to stain nuclei. Scale bar = 25 μm.

(B) Frequency of cells positive for Tbc1d24 cytoophidium in Neuro2a cells treated with zeocin for 0, 48, 72 and 96 hrs. n.s. not significant. $^{**}p < 0.01$; Student's *t*-test. The data were presented as the means ± SEM.

(C) Length (μm) of Tbc1d24 cytoophidium in Neuro2a cells treated with zeocin for 0, 48, 72 and 96 hrs. $^{**}p < 0.01$; Student's *t*-test. The data were presented as the means ± SEM.

[Results] Line 333 -.

Kinetics for zeocin treatment-induced cytoophidia were analyzed by the time course experiment as follows. Longer incubation with zeocin resulted in more obvious cellular shape changes that were accompanied by more frequent (**S7A and S7B Fig**) and longer (**S7A and S7C Fig**) cytoophidia formation, indicating the positive correlation of the juvenescence loss and cytoophidia formation.

[Methods] Line 211 -.

The length (μm) of cytoophidia was measured using Fiji [39].

For Fig 5, it will be useful to show the levels of siGm14230 in addition to Tbc1d24 for zeocin treatment.

We tested the effect of *Tbc1d24* depletion on si*Gm14230*-induced cellular senescence. As a result, the cell growth arrest and cell death were synergistically augmented by the simultaneous depletion of *Gm14230* and *Tbc1d24*.

We added this result as **S6 Fig**.

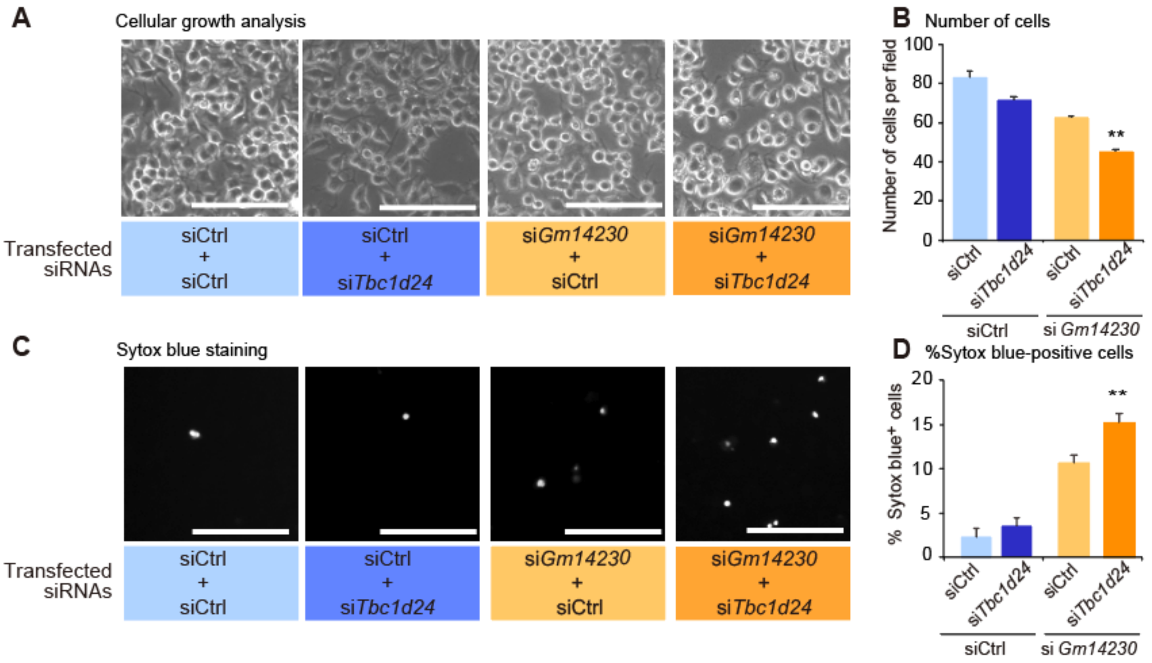

[Figure Legends] Line 741 -.

S6 Fig. The protective effect of Tbc1d24 in the *Gm14230*-depletion-induced loss of cellular juvenescence.

(A) Appearance of Neuro2a cells 48 hrs after the simultaneous knockdown of *Gm14230* and *Tbc1d24*. Indicated siRNAs were transfected in Neuro2a cells for the evaluation of cell growth. Scale bar = 100 μm.

(B) Quantification of Neuro2a cells transfected the indicated siRNAs.

(C) Sytox blue staining in the same fields as in (A). The gray dots indicated dead cells stained by Sytox blue.

(D) Frequency of Sytox blue-positive cells.

n.s. not-significant, $^{**}p < 0.01$; Student's *t*-test. The data were presented as the means ± SEM.

[Results] Line 320 -.

To further assess the resistive role of Tbc1d24 in the juvenescence loss, we next asked the effect of *Tbc1d24* suppression in the context of *Gm14230* depletion. Simultaneous knockdown of *Gm14230* and *Tbc1d24* resulted in synergistic effects on the cell growth impairment (**S6A and S6B Fig**) and cell toxicity (**S6C and S6D Fig**), corroborating that Tbc1d24 constitutes a protective machinery against the juvenescence loss.

[Methods] Line 230 -.

To assess a role of Tbc1d24 in loss of cellular juvenescence induced by *Gm14230* depletion, Neuro2a cells were transfected simultaneously with *Tbc1d24* and *Gm14230* siRNAs. The cell growth and viability were analyzed 48 hrs after the transfection. For the viability assessment, Sytox blue staining was performed described as above.

Finally, it is useful to show the half-life of these proteins with formation of cytoophidia.

To assess the half-life of Tbc1d24 protein in the conditions inducing cytoophidia formation, we performed western blot analysis with Tbc1d24 in the presence of translation-inhibiting agent cycloheximide (CHX). As a result, the zeocin treatment elongated the half-life of Tbc1d24 protein upon cytoophidia formation. Therefore, forming cytoophidia are suggested as storage of Tbc1d24 to protect protein degradation.

We added this result as **S8 Fig**.

We also added description in **Results** and **Methods** as below.

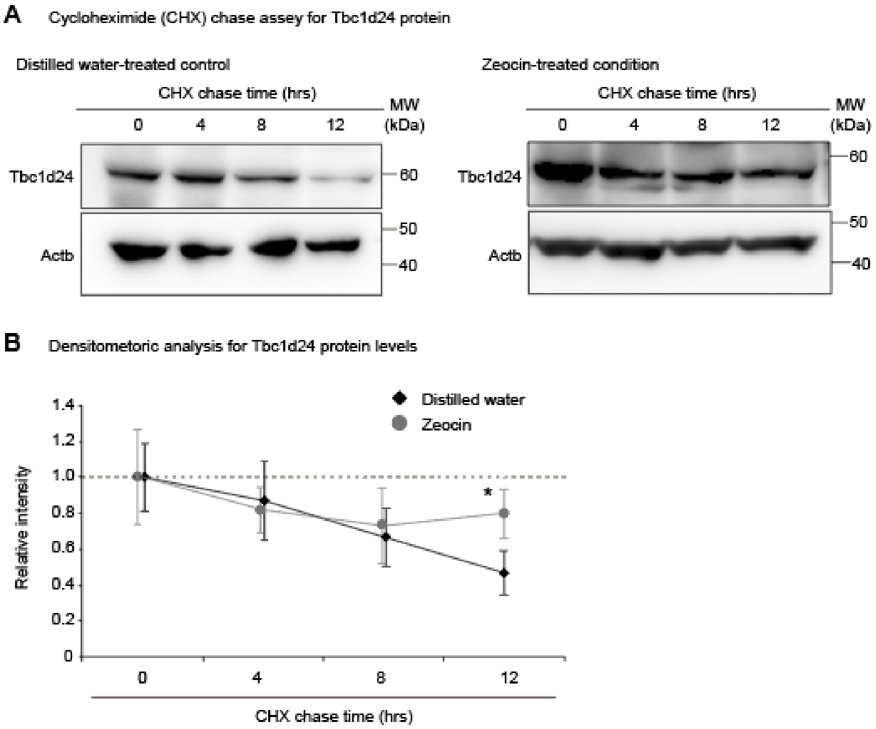

[Figure legends] Line 762 -.

S8 Fig. Tbc1d24 protein is stabilized by cytoophidia formation.

(A) The half-life of Tbc1d24 protein was analyzed by the cycloheximide (CHX) chase assay in Neuro2a cells treated with control distilled water or zeocin for 72 hrs. The cells were treated with CHX 20 μg/ml for 0, 4, 8 and 12 hrs, respectively. Actb was used as a loading control.

(B) Quantitative analysis of western blots for Tbc1d24 was shown. The intensity of the bands was quantified and normalized to those of Actb. The ratios to Actb were further normalized to 0 hr. *$p < 0.05$; Student's *t*-test. The data were presented as the means ± SEM.

[Results] Line 337 -.

The finding of time-dependent increment of the cytoophidia led us to a question whether the Tbc1d24 protein half-life was affected by the cytoophidia formation. The cycloheximide chase assay for Tbc1d24 protein showed elongated half-life of Tbc1d24 protein in zeocin-treated cells compared to control cells, suggesting the enhanced stability of protein contributed to the larger cytoophidia (**S8 Fig**).

[Methods] Line 174 -

Cycloheximide chase assay

To access whether the cytoophidia formation affected the half-life of Tbc1d24 protein, the Tbc1d24 protein expression levels were analyzed in Neuro2a cells treated with zeocin at 200 µg/ml or control distilled water for 72 hours in the presence of cycloheximide (CHX) at 20 µg/ml. Cell lysates were prepared at 0, 4, 8, 12 hours after the CHX treatment and analyzed for the expression of Tbc1d24 protein by western blots as described in above.

Thank you for giving us the precious opportunity to significantly improve our paper and refine the research constructively.
